# ﻿Phylogenomics of North American cybaeid spiders (Araneae, Cybaeidae), including the description of new taxa from the Klamath Mountains Geomorphic Province

**DOI:** 10.3897/zookeys.1226.140204

**Published:** 2025-02-06

**Authors:** Marshal Hedin, Martín J. Ramírez, Rodrigo Monjaraz-Ruedas

**Affiliations:** 1 Department of Biology, San Diego State University, San Diego, California 92182–4614, USA San Diego State University San Diego United States of America; 2 Division of Arachnology, Museo Argentino de Ciencias Naturales - CONICET, Buenos Aires, Argentina Division of Arachnology, Museo Argentino de Ciencias Naturales Buenos Aires Argentina; 3 Natural History Museum of Los Angeles County, 900 Exposition Boulevard, Los Angeles, California 90007, USA Natural History Museum of Los Angeles County Los Angeles United States of America

**Keywords:** California, mountains, short-range endemics, taxonomy, ultraconserved elements

## Abstract

The systematics of humble-in-appearance brown spiders (“marronoids”), within a larger group of spiders with a modified retrolateral tibial apophysis (the RTA Clade), has long vexed arachnologists. Although not yet fully settled, recent phylogenomics has allowed the delimitation and phylogenetic relationships of families within marronoids to come into focus. Understanding relationships within these families still awaits more comprehensive generic-level sampling, as the majority of described marronoid genera remain unsampled for phylogenomic data. Here we conduct such an analysis in the family Cybaeidae Banks, 1892. We greatly increase generic-level sampling, assembling ultraconserved element (UCE) data for 18 of 22 described cybaeid genera, including all North American genera, and rigorously test family monophyly using a comprehensive outgroup taxon sample. We also conduct analyses of traditional Sanger loci, allowing curation of some previously published data. Our UCE phylogenomic results support the monophyly of recognized cybaeids, with strongly supported internal relationships, and evidence for five primary molecular subclades. We hypothesize potential morphological synapomorphies for most of these subclades, bringing a robust phylogenomic underpinning to cybaeid classification. A new cybaeid genus *Siskiyu***gen. nov.** and species *Siskiyuarmilla***sp. nov.** is discovered and described from far northern California and adjacent southern Oregon and a new species in the elusive genus *Cybaeozyga*, *C.furtiva***sp. nov.**, is described from far northern California.

## ﻿Introduction

Phylogenomic datasets collected over the past decade have allowed the backbone of spider phylogeny to come into focus. Major clades such as Synspermiata, Araneoidea, and the retrolateral tibial apophysis (RTA) Clade, which also have morphological support (e.g., Griswold et al. 1999, 2005; Michalik and Ramírez 2014), are now consistently recovered in multiple phylogenomic analyses (Garrison et al. 2016; Ramírez et al. 2021; Kulkarni et al. 2021; Shao et al. 2023; Zhang et al. 2023). Similarly, the RTA Clade includes several supported subclades, including the Dionycha, Oval Calamistrum Clade (OCC), and the “marronoid” clade. Within these core RTA subclades, phylogenetic relationships become less certain, as phylogenomic datasets strive to catch up with the immense taxonomic diversity that these clades encompass. Not only are family-level interrelationships within these RTA subclades unsettled to various degrees, but the definition and composition of included families continues to undergo revision (e.g., Azevedo et al. 2022; Gorneau et al. 2023; Kelly et al. 2023; Kulkarni et al. 2023).

**Figure 1.**
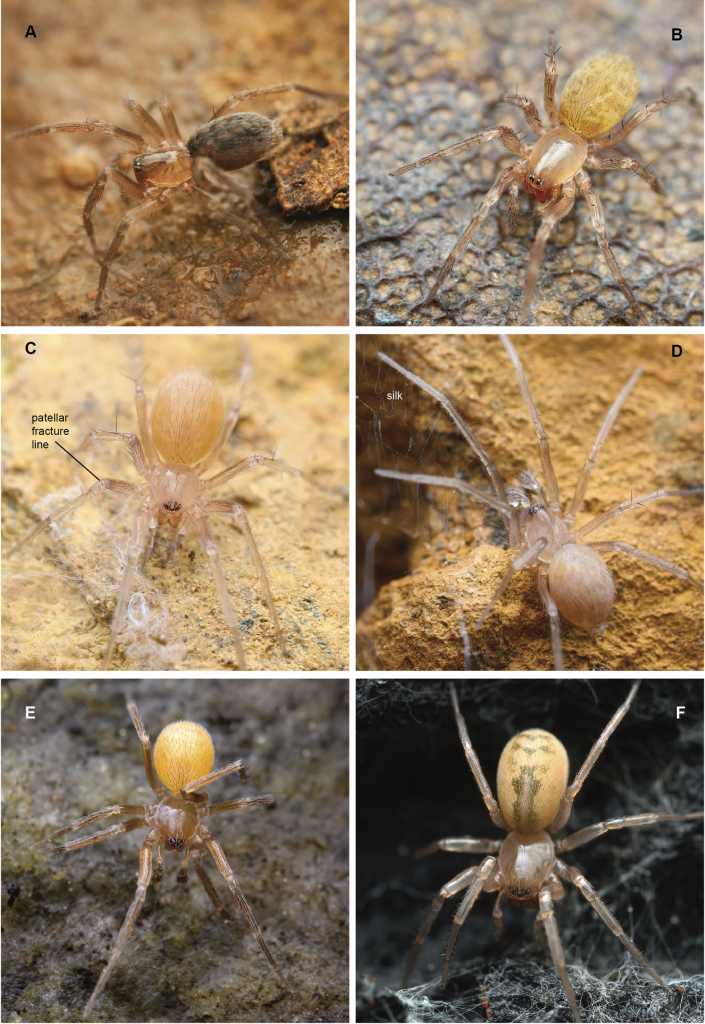
Images of live cybaeid spiders. **A***Neocryphoecabeattyi* ♀, Pinal County, AZ, RWM 22_011 **B***Blabomma sp* ♀, San Diego County, CA, MCH 22_020 **C***Siskiyuarmilla* sp. nov. immature ♀, Siskiyou County CA, MCH 24_057; patellar fracture line labeled **D***Siskiyuarmilla* sp. nov. immature ♂, Siskiyou County CA, MCH 24_057; with silk webbing presumed to belong to the spider **E***Cybaeotashastae* ♀ Chamberlin & Ivie, 1937, East Rosebud Creek, MT **F***Cybaeussomesbar* ♀ Bennett, 2009, Shasta County, CA, MCH 24_012. All images taken by M Hedin, except for *Neocryphoeca* (RW Mendez).

One such taxonomically dynamic group includes a large lineage of RTA Clade spiders called the “marronoids”. Marronoids were recognized following analyses of the Sanger sequence datasets of Wheeler et al. (2017), comprising a molecular clade of “brown spiders” (Fig. 1) which then lacked morphological synapomorphies. Both earlier (Spagna and Gillespie 2008; Miller et al. 2010; Spagna et al. 2010), and subsequent (Crews et al. 2020) Sanger-based studies were important contributions in marronoid molecular systematics. The recent phylogenomic study of Gorneau et al. (2023) took another large step forward in resolving marronoid phylogeny and classification by combining extensive taxon sampling with phylogenomics based on the capture of ultraconserved element (UCE) loci. These phylogenomic results led to the elevation of two new families, and the redefinition and re-circumscription of several others (reconciling current taxonomy with well supported tree topologies), for a marronoid clade which now includes eleven families. Phylogenomic research based on dense sampling of austral marronoids has likewise recovered many novel family-level lineages that await formal delimitation (Kelly et al. 2023).

One of the eleven families redefined by Gorneau et al. (2023) included the Cybaeidae. The complicated taxonomic history of cybaeids was comprehensively summarized by these authors (e.g., Gorneau et al. 2023: fig. 2), and is not repeated here. Following from predecessors (Bennett 1991), the cybaeids are morphologically defined as spiders possessing three claws, a single row of tarsal trichobothria, uniquely configured posterior lateral spinnerets and lacking a cribellum (Gorneau et al. 2023). Although their phylogenomic results were based on UCEs available for five cybaeid genera, morphological similarities and other prior systematic studies led the authors to include 20 total genera in the family. Three other genera have since been added, including *Guicybaeus*Wang et al., 2023, *Neocybaeina* Bennett, 2023 and *Rothaeina* Bennett, 2023 (World Spider Catalog 2024). As of late-2024 the family includes 23 genera and 302 species (Table 1; World Spider Catalog 2024), although we question the inclusion of *Vagellia* Simon, 1899, from Sumatra (see also Lehtinen 1967).

**Figure 2.**
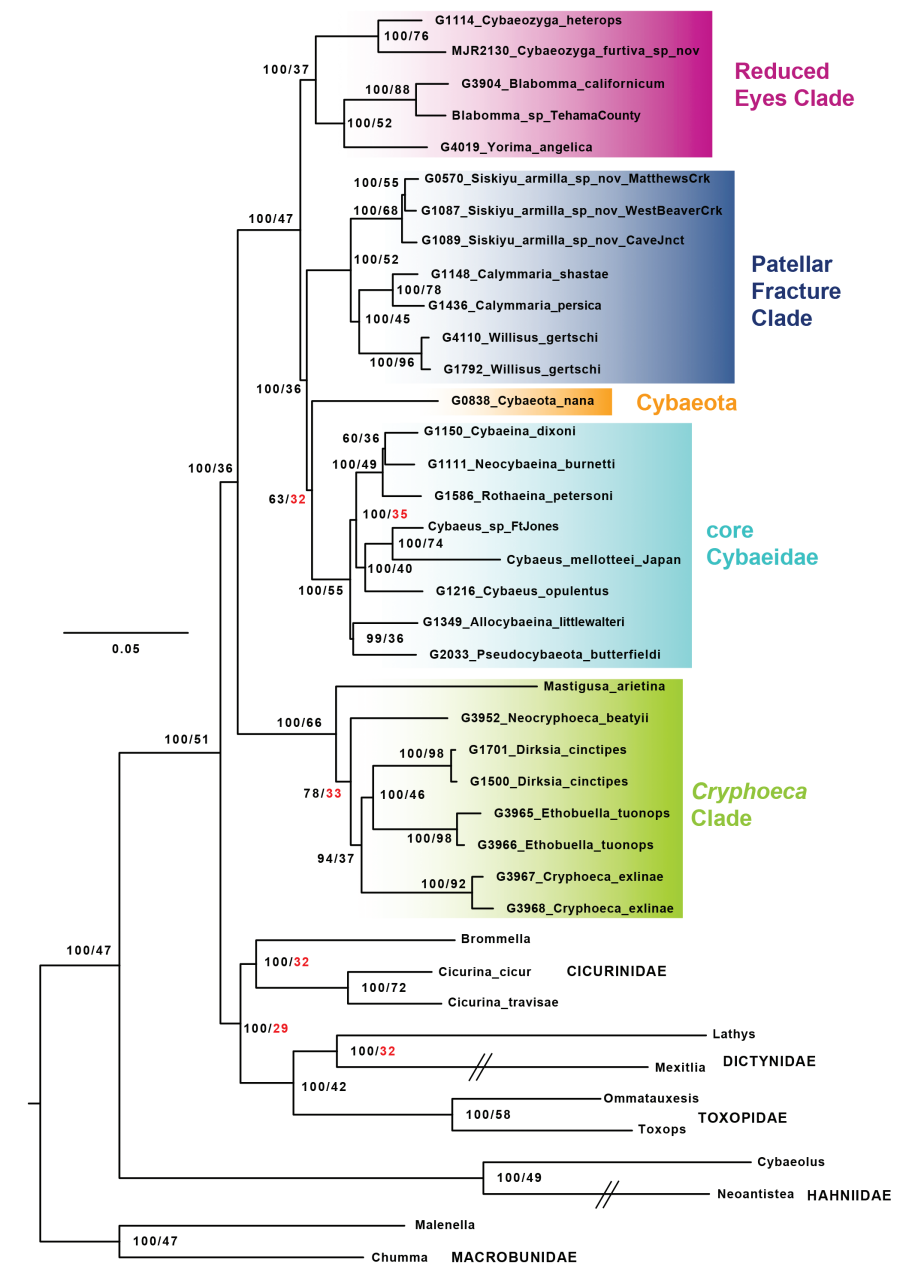
UCE concatenated IQTree tree from 50p_filtered matrix. Nodal values correspond to bootstrap / siteCF (rounded to nearest integer). Low (below 35%) siteCF values highlighted in red. Specimen numbers correspond to those in Suppl. material 1. Outgroup branches with hashes truncated for graphical purposes. Primary cybaeid subclades labeled.

**Table 1.** Described cybaeid genera, following the World Spider Catalog (2024), including number of described species and general geographic distribution. Unsampled taxa denoted with an asterisk.

	LSID	Described species	Distribution
**core Cybaeidae**			
*Allocybaeina* Bennett, 2020	[urn:lsid:nmbe.ch:spidersp:052294]	monotypic	northwestern CA
*Cybaeina* Chamberlin & Ivie, 1932	[urn:lsid:nmbe.ch:spidergen:03378]	3 species	western Nearctic
*Cybaeus* L. Koch, 1868	[urn:lsid:nmbe.ch:spidergen:03381]	198 species	Southern Europe, Japan, Korea, Nearctic
*Guicybaeus Wang, Chen, Yang & Zhang, 2023	[urn:lsid:nmbe.ch:spidersp:059463]	monotypic	China
*Neocybaeina* Bennett, 2023	[urn:lsid:nmbe.ch:spidergen:06067]	2 species	Oregon
*Pseudocybaeota* Bennett, 2022	[urn:lsid:nmbe.ch:spidergen:05586]	3 species	western Nearctic
*Rothaeina* Bennett, 2023	[urn:lsid:nmbe.ch:spidergen:06068]	5 species	western Nearctic
*Sincybaeus Wang & Zhang, 2022	[urn:lsid:nmbe.ch:spidersp:056655]	4 species	China, Japan
**Reduced Eyes Clade**			
*Blabomma* Chamberlin & Ivie, 1937	[urn:lsid:nmbe.ch:spidergen:01967]	11 species	western Nearctic, Korea
*Cybaeozyga* Chamberlin & Ivie, 1937	[urn:lsid:nmbe.ch:spidersp:021338]	2 species	western Nearctic
*Symposia Simon, 1898	[urn:lsid:nmbe.ch:spidergen:03384]	6 species	Columbia and Venezuela
*Yorima* Chamberlin & Ivie, 1942	[urn:lsid:nmbe.ch:spidergen:02003]	6 species	western Nearctic, Cuba?
**Patellar Fracture Clade**			
*Calymmaria* Chamberlin & Ivie, 1937	[urn:lsid:nmbe.ch:spidergen:01935]	31 species	Nearctic
*Siskiyu* gen. nov.	–	monotypic	northern California
*Willisus* Roth, 1981	[urn:lsid:nmbe.ch:spidersp:022044]	monotypic	California
**Cryphoeca Clade**			
*Cryphoeca* Thorell, 1870	[urn:lsid:nmbe.ch:spidergen:01936]	13 species	Southern Europe, Japan, Nearctic
*Cryphoecina Deltshev, 1997	[urn:lsid:nmbe.ch:spidersp:021881]	monotypic	Montenegro
*Dirksia* Chamberlin & Ivie, 1942	[urn:lsid:nmbe.ch:spidergen:01939]	2 species	western Nearctic, France
*Ethobuella* Chamberlin & Ivie, 1937	[urn:lsid:nmbe.ch:spidergen:01940]	2 species	western Nearctic
*Mastigusa* Menge, 1854	[urn:lsid:nmbe.ch:spidergen:01985]	4 species	Europe, Turkey, Russia (Europe, Caucasus, South Siberia), Georgia, Iran
*Neocryphoeca* Roth, 1970	[urn:lsid:nmbe.ch:spidergen:01949]	2 species	Arizona
*Tuberta Simon, 1884	[urn:lsid:nmbe.ch:spidergen:01954]	2 species	Britain, France, Switzerland, Belgium, Germany, Croatia, Bulgaria, Turkey, Azerbaijan
***Cybaeota***			
*Cybaeota* Chamberlin & Ivie, 1933	[urn:lsid:nmbe.ch:spidergen:03379]	5 species	Russia, Nearctic
**Dubious Cybaeids**			
*Vagellia Simon, 1899	[urn:lsid:nmbe.ch:spidersp:021452]	monotypic	Sumatra

Cybaeid spiders show apparent niche conservatism (Wiens and Graham 2005) and a general microhabitat preference for living on or beneath rocks and woody debris in shaded, cool forested habitats (Roth and Brame 1972; Bennett et al. 2017). Other microhabitats include caves, ant nests, and moss on tree trunks (Bennett 1988; Castellucci et al. 2024). Many cybaeid genera are notably species rich with arrays of short-range endemic species (following Harvey et al. 2011), including genera such as *Cybaeus* L. Koch, 1868, *Blabomma* Chamberlin & Ivie, 1937, and *Calymmaria* Chamberlin & Ivie, 1937 (Heiss and Draney 2004; Bennett et al. 2021, 2022a). Five described genera are monotypic, rare in collections, and very poorly known, including *Allocybaeina* Bennett, 2020, *Cryphoecina* Deltshev, 1997, *Cybaeozyga* Chamberlin & Ivie, 1937, *Guicybaeus*, and *Willisus* Roth, 1981. More broadly, cybaeids appear to have a classic Holarctic disjunct biogeography, with centers of diversification in eastern and western North America, southeast Asia, and the Russian Far East, and in southern Europe extending eastwards. Such a disjunct continental distribution has been described for many plant and animal lineages, including other arachnids and spiders (Schönhofer et al. 2013, Derkarabetian et al. 2018, Ledford et al. 2021). While most cybaeid genera occupy a single continental region (Table 1), some genera are hypothesized to span continents, such as *Cybaeota* Chamberlin & Ivie, 1933 from western North America and Russia (Marusik et al. 2020), and *Dirksia* Chamberlin & Ivie, 1942 from western North America and France (Lehtinen 1967). *Cybaeus* and *Cryphoeca* Thorell, 1870 include described species in all Holarctic regions.

Here we use the phylogenomic results of Gorneau et al. (2023) as a foundation and springboard to further understand cybaeid generic-level relationships. Using a large outgroup taxon sample, we formally test the monophyly of the family, as other recent phylogenomic analyses have not always recovered this monophyly (Kulkarni et al. 2023). Within the family we greatly increase generic-level sampling by including UCE-based phylogenomic data for 17 genera (of 22 total), extending from the five genera of Gorneau et al. (2023). This ingroup sampling includes all described North American genera, European taxa from prior studies (including *Mastigusa* Menge, 1854) but lacked Asian taxa. Western North America has the greatest described generic diversity, and as such, could represent the center of origin for the family. This taxon sample also allows a preliminary test of monophyly for some species-rich genera, including *Cybaeus*. Our well-supported phylogenomic results show that recognized cybaeids are monophyletic, with strongly supported internal relationships, bringing a robust phylogenomic underpinning to cybaeid classification. We provide evidence for five primary subclades in the sampled taxa and hypothesize potential morphological synapomorphies for most of these molecular subclades. We discover and describe a new cybaeid genus, *Siskiyu* gen. nov., and species, *Siskiyuarmilla* sp. nov., from far northern California and adjacent southern Oregon, and a new species in the rare genus *Cybaeozyga*, *C.furtiva* sp. nov., from far northern California.

## ﻿Materials and methods

### ﻿Taxon sampling

Prior phylogenomic studies have recovered a well-supported subclade of six marronoid families, related as follows: (Macrobunidae, (Hahniiidae, (Cybaeidae, (Cicurinidae, (Toxopidae, Dictynidae))))); Gorneau et al. 2023; see also Kulkarni et al. 2023). We used previously published and original phylogenomic data for multiple genera for each of the five outgroup families from above (Suppl. material 1), allowing a robust test of cybaeid monophyly.

Most cybaeid phylogenomic data included here were derived from our original collections, although we also included a handful of ingroup samples from prior studies (Suppl. material 1). This included data for *Mastigusa*. Recent Sanger analyses support this genus as a cybaeid (Castellucci et al. 2023), but placement in phylogenomic analyses has been unstable (Gorneau et al. 2023, Kulkarni et al. 2023). We were able to sample and include original data for all described North American cybaeid genera (Suppl. material 1). Several rare genera were collected from their respective type localities, including *Cybaeozyga*, *Neocryphoeca* Roth, 1970, and *Willisus* (see Suppl. material 1). Our original sample also includes the important genera *Ethobuella* Chamberlin & Ivie, 1937 and *Dirksia*, which are listed as cybaeids by Gorneau et al. (2023) but lack phylogenomic data. Phylogenetic analyses of published Sanger data consistently place these two genera outside of Cybaeidae (e.g., Crews et al. 2020; Kulkarni et al. 2023; Castellucci et al. 2023).

Spiders were searched for in appropriate microhabitats and collected by hand or with an aspirator. Most spiders were preserved in the field in either 80% EtOH for morphological study, or in 100% EtOH for DNA analysis. Spiders were identified to genus using available keys (Bennett et al. 2017), and to the species level using the relevant taxonomic literature (Chamberlin and Ivie 1937; Roth 1956, 1970, 1981; Bennett 1988; Heiss and Draney 2004; Bennett et al. 2020, 2022b, 2023). Images of genitalia and/or entire spiders for voucher specimens used in UCE experiments are available on ecdysis (https://ecdysis.org); specimens are currently housed in the SDSU Terrestrial Arthropods Collection (SDSU_TAC).

### ﻿UCE data collection and processing

Genomic DNA was extracted from leg tissues using the DNeasy Kit (Qiagen GmbH, Hilden, Germany), with at least 250 ng of genomic DNA used for UCE library preparation. UCE library preparation and library sequencing were performed at RAPID Genomics, with target enrichment performed using the Spider 2Kv1 (Kulkarni et al. 2020) or RTA Spider Clade probeset (Zhang et al. 2023).

Bioinformatic analyses were conducted on the Mesxuuyan HPC at SDSU. Raw demultiplexed reads were quality-filtered and cleaned of adapter contamination with Trimmomatic (Bolger et al. 2014), using parameters PE ILLUMINACLIP:$adaptersfasta:2:30:10:2:keepBothReads LEADING:5 TRAILING:15 SLIDINGWINDOW:4:15 MINLEN:40. Cleaned reads were assembled into contigs using SPADES v3.13.0 (Prjibelski et al. 2020) with the --isolate option. PHYLUCE (Faircloth 2016) was used to map and identify UCE loci, mapping contigs against the RTA Spider Clade probeset (Zhang et al. 2023) using default (80, 80) matching values.

We also extracted UCEs from published low-coverage whole genomes and transcriptomes in-silico using PHYLUCE (https://phyluce.readthedocs.io/en/latest/tutorials/tutorial-3.html). Raw reads were downloaded from the SRA (Suppl. material 1), cleaned, and assembled using the same parameters as with the UCE data. Default parameters were used across the entire process except for coverage and identity parameters in “phyluce_probe_run_multiple_lastzs_sqlite”, set at 80, 80 to maintain consistent matching values across all data types.

Individual UCE loci were aligned, trimmed, and filtering using FUSe (https://github.com/rmonjaraz/FUSe; Monjaraz-Ruedas et al. 2024). This included aligning with the MAFFT globapair option (Katoh et al. 2013), and trimming using trimAl (Capella-Gutiérrez et al. 2009) with the -automated1 option. We also removed highly divergent sequences (60%) (--remove-div -d 0.6) and sequences shorter than 70% of the total alignment length (--remove-short -s 0.7), retaining alignments with a minimum of 4 sequences and longer than 50bp. Subsequent 50% and 80% completeness matrices were created (--get-completeness -e 0.8 and -e 0.5). Finally, we manually curated the above alignments in Geneious Prime 2023, removing any remaining obviously divergent individual sequences, and matrices with an average pairwise identity below 80%. These matrices were named 50p_filtered and 80p_filtered, respectively.

Because several outgroup taxa revealed high sequence divergences (see also Gorneau et al. 2023), with phylogenetic placement and estimated branch lengths possibly impacted by filtering and sequence trimming method, we implemented an alternative trimming workflow by combining PhyIN (Maddison 2024) and FUSe. This workflow consisted of aligning with MAFFT using the globalpair option, followed by removal of highly divergent sequences (70% divergence) (--remove-div -d 0.7), trimming of gaps using a Simple Gappiness Filter (sgp.py) (Maddison 2024) with options -gS -gB -t -1 in combination with PhyIN using options -b 10 -d 2 -p 0.5 -e. Finally, after trimming, remaining short sequences were removed from alignments using FUSe (--remove-short -s 0.7) and 50 and 80% completeness matrices were created. These matrices were named 50p_PhyIN and 80p_PhyIN, respectively.

### ﻿“Sanger Loci” data

We extracted traditionally used Sanger loci (18S, 28S, H3) from raw reads and compared these to previously published Sanger data for various cybaeid taxa (Suppl. material 1). Our primary objective here was to better understand the phylogenetic behavior of Sanger sequences for particular taxa (e.g., *Ethobuella* and *Dirksia*, see Crews et al. 2020; Kulkarni et al. 2023; Castellucci et al. 2023), given our larger taxon sample, available voucher specimens, and well-resolved phylogenomic backdrop.

Sanger loci were harvested from UCEs, low-coverage genomes and transcriptome data using custom scripts (“loci_byCatch.sh”, on Dryad at https://doi.org/10.5061/dryad.2v6wwpzz4). A reference fasta file (“reference_cyb.fasta”, on Dryad) containing target loci was created from previously published ingroup data. Cleaned fastq files were mapped against this reference using BWA (Li 2013). Resulting BAM files were used for calling consensus sequences for each sample and locus using the consensus function of SAMTOOLS v1.16 (Danecek et al. 2021); for each mapped sample we retained the longest sequence for downstream analysis. Consensus sequences were merged, aligned, and trimmed with FUSe using the MAFFT globalpair option for aligning and trimAl -automated1 option for trimming, followed by alignment inspection in Geneious Prime 2023.

### ﻿Phylogenetic analyses

For concatenated UCE and Sanger matrices, and for individual Sanger loci matrices, we conducted maximum likelihood analyses with IQ–TREE 2 (Minh et al. 2020a). Concatenated analyses included individual loci as separate partitions, with 1000 replicates of ultrafast bootstrapping and optimal model search using ModelFinder (Kalyaanamoorthy et al. 2017). For 50p_filtered and 80p_filtered UCE matrices, we also estimated gene trees from individual alignments and calculated gene (gCF) and site (sCF) concordance factors (Minh et al. 2020b) using IQTree; sCF values were estimated with the likelihood option --scfl (Mo et al. 2023).

For 50p_filtered and 80p_filtered UCE matrices only, species trees were also estimated under a multispecies coalescent model using weighted ASTRAL (wASTRAL, Zhang and Mirarab 2022). Input gene trees for wASTRAL were estimated using IQ–TREE 2 with 1000 replicates of ultrafast bootstrapping and treated as unrooted. We used the wASTRAL hybrid scheme to weight gene trees using both long terminal branches and weakly-supported nodes. Internal ASTRAL branch lengths were estimated in coalescent units, with branch support measured as local posterior probability values (Sayyari and Mirarab 2016).

### ﻿Taxonomy

Holotype and paratype specimens have been deposited at the San Diego Natural History Museum (SDNHM) and Museo Argentino de Ciencias Naturales (MACN). All other specimens referenced with San Diego State University numbers are housed in the SDSU Terrestrial Arthropods Collection.

Specimen measurements were taken using an eyepiece micrometer at 4× magnification with an Olympus SZX12 stereomicroscope fitted with 10 × ocular lenses (SDSU) or a Leitz stereomicroscope with an eyepiece micrometer on an 8× ocular (MACN). All measurements are reported in millimeters. Appendage measurements were taken from the left appendage. Tarsal claws were examined using a Leica M205C microscope at 16× magnification.

Specimens were digitally imaged using a Visionary Digital BK plus system including a Canon 5D Mark II digital camera and Infinity Optics Long Distance Microscope (SDSU) or a Leica M205 with a DFC 295 digital camera (MACN). Individual images were combined into a composite image using Helicon Focus V6.6.2 software, then edited using Adobe Photoshop. Female spermathecae were dissected from specimens using fine forceps, immersed in BioQuip specimen clearing fluid on a depression slide, then imaged directly in this fluid on slides. Other images were taken with specimens immersed in filtered 70% EtOH, using KY jelly to secure samples.

## ﻿Results and discussion

### ﻿Taxon sampling and data processing

Voucher specimen information for UCE and “Sanger loci” analyses are summarized in Suppl. material 1. Raw read data from original UCE capture experiments have been submitted to the Sequence Read Archive (BioProject ID: PRJNA1183915). Numbers of assembled contigs, and loci in the 50p_PhyIn UCE matrix, are included in Suppl. material 1. Input matrices, analysis log files, and output tree files are on Dryad (https://doi.org/10.5061/dryad.2v6wwpzz4).

### ﻿Phylogenetic results

UCE matrices included data for 10 outgroup genera, and 29 total ingroup samples representing 18 genera (Suppl. material 1). The stricter 80p_filtered and 50p_filtered matrices ranged in concatenated length from 130,545 base pairs (233 loci) to 294,777 base pairs (554 loci), respectively. 80p_PhyIN and 50p_PhyIN matrices ranged in concatenated length from 150,541 base pairs (355 loci) to 344,448 base pairs (874 loci), respectively.

Concatenated maximum likelihood analyses of UCE matrices recover outgroup relationships as expected (Gorneau et al. 2023), and cybaeid monophyly (Fig. 2, Suppl. material 2). Different alignment filtering workflows impacted recovered concatenated branch lengths, but topologies remained largely unchanged (Fig. 2, Suppl. material 2). Bootstrap support values for cybaeid monophyly are consistently high across UCE analyses (99 or 100). Gene tree topological variance, as measured by concordance factors (Lanfear and Hahn 2024), is moderate to high for this node (gene CF = 25, site CF = 36) in comparison to tree-wide values.

Consistent relationships within cybaeids include a *Cryphoeca* Clade (*Mastigusa*, *Dirksia*, *Ethobuella*, *Neocryphoeca*, *Cryphoeca*) sister to all other genera. The deepest branches within the family separate *Cryphoeca* Clade members from other primary lineages. Within the former, *Dirksia* is strongly supported as sister to *Ethobuella*, but other *Cryphoeca* Clade intergeneric relationships are relatively weakly supported.

Sister to the *Cryphoeca* Clade, all remaining genera are grouped into four main subclades, informally named here the Reduced Eyes Clade (REC), Patellar Fracture Clade (PFC), *Cybaeota*, and core Cybaeidae (all further discussed and defined below). Concatenated bootstrap support values in this part of cybaeid phylogeny are generally high, except for the node uniting *Cybaeota* and core cybaeids, and *Neocybaeina* plus *Cybaeina* Chamberlin & Ivie, 1932 within the latter. The *Cybaeota* plus core cybaeid node also has notably low site CF values (site CF = 32, 34), indicating high topological variance at this node (Fig. 2, Suppl. material 2). Concatenated results support *Allocybaeina* sister to *Pseudocybaeota* Bennett, 2022 with high support. We note that the overall five-clade structure for the entire family is reflected in early Sanger loci phylogenetic results of Spagna et al. (2010: fig. 6) and Crews et al. (2020: fig. 1), although these earlier efforts had sparser taxon sampling.

**Figure 3.**
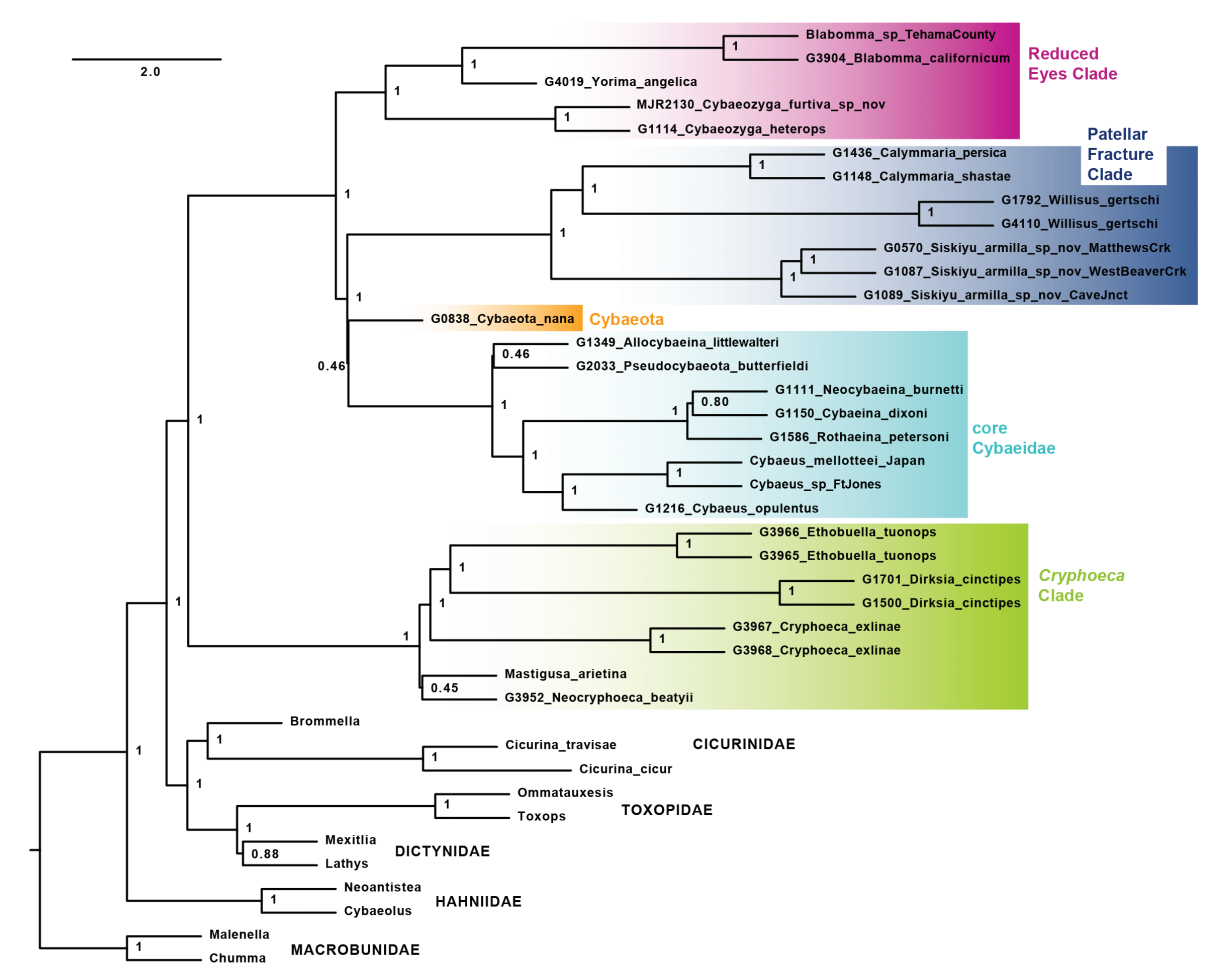
UCE wASTRAL species tree from 50p_filtered matrix, with posterior probability values. Branch lengths in coalescent units for internal branches only, terminal branch lengths arbitrary. Primary cybaeid subclades labeled.

Species trees were estimated under the wASTRAL multispecies coalescent model for 50p_ and 80p_filtered matrices. For the 50p matrices, cybaeid monophyly and five cybaeid subclades are recovered with high posterior probabilities (posterior probability = 1; Fig. 3). Within the *Cryphoeca* Clade, *Mastigusa* is sister to *Neocryphoeca*, but at low posterior probability (pp = 0.45). Likewise, relationships between the PFC, *Cybaeota*, and core Cybaeidae are unresolved (pp = 0.45), and the sister relationship between *Allocybaeina* and *Pseudocybaeota* is poorly supported (pp = 0.45). The 80p_filtered ASTRAL results are broadly similar, but here *Mastigusa* is sister to all other *Cryphoeca* Clade members, eroding the support for this subclade (pp = 0.67; Fig. 3). PFC, *Cybaeota*, and core cybaeid interrelationships are again a trichotomy (pp = 0.47), and *Allocybaeina* is sister to all other core cybaeid members, rather than sister to *Pseudocybaeota*. *Neocybaeina* plus *Cybaeina* is recovered in both ASTRAL analyses with moderate support.

**Figure 4.**
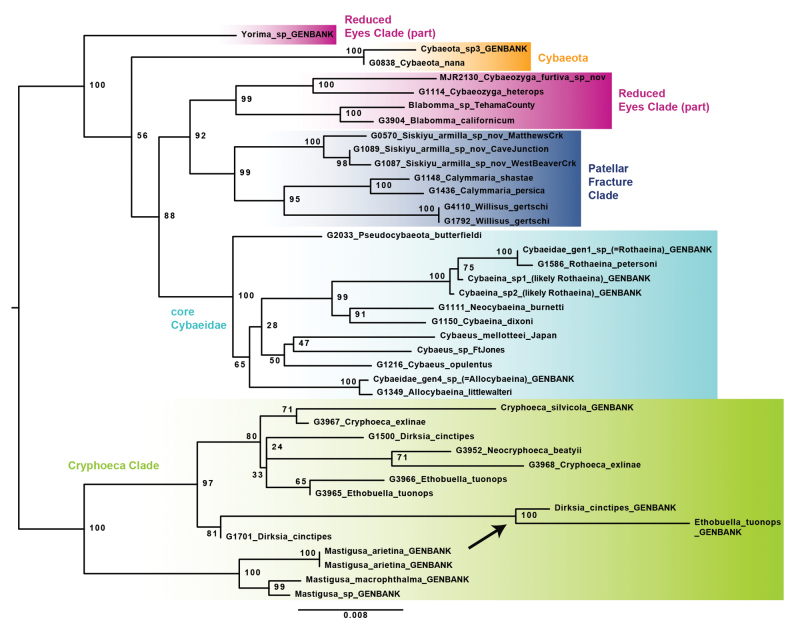
IQTree tree from concatenated 18S, 28S, and H3 Sanger loci, for ingroup samples only. Long branch leading to legacy GenBank data for *Ethobuella* and *Dirksia* at arrow. Primary cybaeid subclades labeled. Specimen numbers correspond to those in Suppl. material 1.

Concatenated analyses of 18S, 28S, and H3 data for ingroup samples separate *Cryphoeca* Clade taxa from others, with the four lineages within the latter mostly recovered (*Yorima* GenBank sequences are placed outside of REC; Fig. 4). These analyses allow curation of some previously published sequences (see Suppl. material 1), and place *Mastigusa* as sister to all other *Cryphoeca* Clade members with high support, contra the recent analyses of Castellucci et al. (2023) where *Mastigusa* is sister to *Cryphoeca*. *Mastigusa* as sister to all other *Cryphoeca* Clade genera also finds moderate support in some UCE topologies (e.g., Fig. 2).

A conspicuously long branch is found within the *Cryphoeca* Clade, separating published *Ethobuella* and *Dirksia* sequences from those recovered from UCE raw read data. These long branches in the concatenated analysis likely reflect long branches also found on H3 and 18S topologies (Suppl. material 2). We suspect that these H3 and 18S sequences are contaminants and may explain the unexpected *Ethobuella* and *Dirksia* placements found in prior studies (Crews et al. 2020: fig. 2; Kulkarni et al. 2023: fig. 15; Castellucci et al. 2023: fig. 2).

### ﻿Morphological synapomorphies for phylogenomic clades

**Figure 5.**
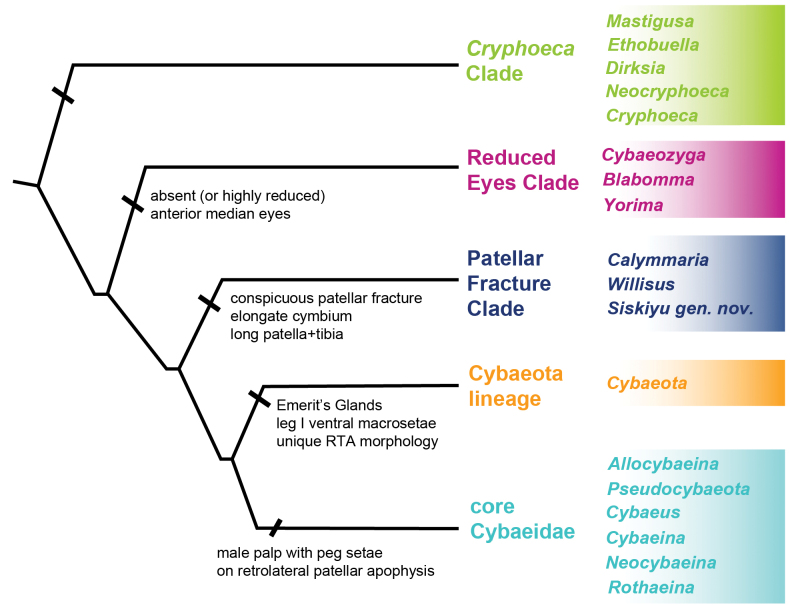
Summary tree with primary cybaeid subclades and sampled subclade genera. Hypothesized diagnostic morphological characters for each subclade (as discussed in the text) summarized.

Below we discuss possible morphological synapomorphies for the five primary phylogenomic subclades recovered within Cybaeidae (summarized in Fig. 5). Two of these groupings have been treated historically as formal subfamilies by some authors, but we here treat them informally, and do not elevate all equivalent clades to the subfamily level. We note that no prior circumscribed subfamily, as originally delimited, is recovered in phylogenomic analyses, further justifying this informal treatment.

We also note here an interesting pattern of unbalanced species diversification, with species-poor lineages sister to particularly species-rich lineages. Examples include *Blabomma* plus *Yorima* Chamberlin & Ivie, 1942, sister to *Cybaeozyga* (~ 50 vs 5 species, many undescribed), *Cybaeus* sister to *Cybaeina* plus relatives (200 vs 10 species), and *Calymmaria* sister to *Willisus* (31 vs 1 species) (Table 1).

*Cryphoeca* Clade – Lehtinen (1967) included *Tuberta* Simon 1884, *Cryphoeca*, *Dirksia*, *Ethobuella*, and *Calymmaria* in his “Cryphoecinae”, a subfamily of his Hahniidae, but was ambiguous regarding defining features for the group. Roth and Brame (1972) later circumscribed a more inclusive “Group Cryphoeceae”, including many taxa now known to belong elsewhere (see below), but noted the morphological similarity of *Neocryphoeca* and *Cryphoeca* (see also Roth 1970). In his unpublished dissertation, Catley (1996) discussed morphological synapomorphies for a clade including *Cryphoeca*, *Tuberta*, *Ethobuella*, and *Calymmaria*, including male palpal tibial modifications and “coxal patches” (Catley 1996: fig. 110a, b). In an unpublished MS thesis, Lo Man Hung (2013) conducted a morphological parsimony analysis of hahniid subfamilies as recognized by Lehtinen (1967), and recovered *Calymmaria* as sister to *Dirksia*, but neither were related to *Neocryphoeca*. Importantly, outgroup sampling in both Catley (1996) and Lo Man Hung (2013) mostly lacked a diversity of cybaeids (e.g., those genera listed below), as cryphoecines at that time were allied with hahniid spiders.

Our *Cryphoeca* Clade essentially follows Lehtinen (1967), including also *Neocryphoeca* and *Mastigusa*, while excluding *Calymmaria* and relatives (see below). *Neocryphoeca*, *Cryphoeca*, *Tuberta*, and *Mastigusa* together share an elaborate male palpal conductor (Castellucci et al. 2024), but this is quite different from the condition found in *Dirksia* and *Ethobuella*. The placement of *Cryphoecina* as a relative of *Cryphoeca*, as hypothesized by Deltshev (1997), should be confirmed; this taxon lacks the elaborate conductor of the latter. The proposed synapomorphies of Catley (1996) do not apply, as his clade also included the distantly related *Calymmaria*. We are thus not aware of morphological synapomorphies applicable to the entire *Cryphoeca* Clade.

Reduced Eyes Clade (REC) – A clade of three genera (*Cybaeozyga*, *Blabomma*, and *Yorima*) including spiders with absent or highly reduced anterior median eyes. This condition is also found in close relatives outside of the family (e.g., some *Lathys* Simon, 1885, *Brommella* Tullgren, 1948, *Cicurina* Menge, 1871) but is elsewhere uncommon in cybaeids. REC members are otherwise morphologically heterogeneous. *Blabomma* and *Yorima* have historically resided in the larger “Group Cryphoeceae” (Roth and Brame 1972), often allied with *Cicurina*. These genera share simple circular female spermathecae but differ in male palpal and spinneret morphology. *Cybaeozyga* is a traditional core cybaeid, but because this genus is elusive and poorly known, has never been comprehensively treated (see Bennett et al. 2023). *Cybaeozyga* possess shorter, more contiguous, anterior spinnerets (see below). UCE concatenated branch lengths separating *Cybaeozyga* from *Blabomma* and *Yorima* are relatively long (Fig. 2, Suppl. material 2), and further analyses with increased sampling might ultimately distinguish these as two distinct groups. Overall, this clade needs revision, with many undescribed species; this revisionary work may serve to find more convincing morphological synapomorphies. The genus *Symposia* Simon, 1898 from South America also includes six-eyed species (see Müller and Heimer 1988), not unlike *Yorima*, and may reside (surprisingly) in this otherwise north temperate clade.

Patellar Fracture Clade (PFC) – A clade including *Calymmaria*, *Willisus*, and *Siskiyu* gen. nov. which share slender legs, a relatively long patella-tibia I, an elongate male cymbium (homoplastic outside of the family), and a distinct fracture line near the base of leg patella (Fig. 1; Roth 1981: fig. 1; Heiss and Draney 2004: figs 3, 134). As discussed in Roth (1981), this fracture line allows legs to drop easily or “autospasize” from this suture. Both Roth (1994) and Heiss and Draney (2004) note that patellar cleavage is also sometimes found in other cybaeids, including *Ethobuella*, REC genera (including a new *Cybaeozyga* species, see below), *Cybaeota*, and *Cybaeina*. As noted by Roth (1994), some of these latter genera autospasize “uncommonly”. We hypothesize an obvious patellar fracture line as a synapomorphy for the PFC; further study in the family is needed to understand if this character circumscribes a broader clade.

*Cybaeota* - Bennett (1988) cites genitalic apomorphies for male *Cybaeota*, including a unique RTA morphology (Bennett 1988: fig. 17), in comparison to other core Cybaeidae (see below). Marusik et al. (2020) noted how male *Cybaeota* palps are distinct from other core Cybaeidae, perhaps more like *Calymmaria* (consistent with unresolved ASTRAL relationships between the PFC, *Cybaeota*, and core Cybaeidae, Fig. 3). *Cybaeota* species also possess conspicuous pairs of ventral tibial and metatarsal macrosetae on anterior legs, which we hypothesize are homoplastic with similar macrosetae found in *Cybaeina* and relatives (and sometimes more distantly related spiders). Bennett (1989) documented Emerit’s Glands, broadly distributed on the integument and possibly producing repugnatorial secretions, in Nearctic species of *Cybaeota*. These glands were also found in *Cybaeotawesolowskae* from the Russian Far East (Marusik et al. 2020: fig. 2H). In Bennett’s (1989) surveys of cybaeid relatives, including representatives of all other primary lineages (including *Dirksia*, *Cryphoeca*, *Blabomma*, *Calymmaria*, *Cybaeina*, and various *Cybaeus*), he failed to find these glands. We hypothesize the combined possession of genitalic, leg macrosetae, and Emerit’s Glands as morphological synapomorphies for a distinct *Cybaeota* lineage.

Core Cybaeidae – with morphological synapomorphies as described by Bennett (1991). In particular, the male palpal retrolateral patellar apophysis includes peg setae (e.g., Bennett et al. 2023: figs 8–13), which we hypothesize have been secondarily reduced or lost in *Pseudocybaeota* (see also Bennett 1991). Internal relationships recovered here show similarities to morphological hypotheses of Bennett (1991: fig. 623), including consistent recovery of the *Cybaeina* subclade, with members (*Cybaeina*, *Neocybaeina*, *Rothaeina*) possessing conspicuous ventral tibial and metatarsal leg macrosetae. Within *Cybaeus*, UCE data recover a member of the California clade (*C.opulentus* Bennett, 2021) as sister to a clade including an unidentified *Cybaeus* from far northern CA (CASENT9030871) plus *C.mellotteei* (Simon, 1886) from Japan. We hypothesize that both latter taxa are members of the Holarctic Clade, as recovered in Sanger loci analyses (Copley et al. 2009; Sugawara et al. 2024).

Recent Sanger loci analyses including putative *Sincybaeus* Wang & Zhang, 2022 have placed this genus within a core cybaeid clade, possibly sister to *Allocybaeina* (Sugawara et al. 2024); these authors also extend the known distribution of *Sincybaeus* to include China, Japan, and possibly Korea. The affinities of *Guicybaeus* from China remain unclear. The six-eyed *Guicybaeus* appears to have peg setae (Wang et al. 2023: fig. 3C), indicating inclusion in core Cybaeidae, but also has conspicuously long posterior spinnerets.

### ﻿Taxonomy

#### ﻿Genus Siskiyu gen. nov.

Taxon classificationAnimaliaAraneaeCybaeidae

D143EFD9-B161-58E1-A385-DBA7ACE1A587

https://zoobank.org/D0905AEA-AFE9-4315-9C43-64705231957A

Figs 1 , 6 , 7

##### Etymology.

A modification of siskiyou, from the Klamath-Siskiyou Mountains that encompass much of the known distribution of this genus. The etymology of siskiyou, possibly indigenous, remains uncertain. Grammatical gender treated as feminine.

##### Diagnosis.

**Figure 6.**
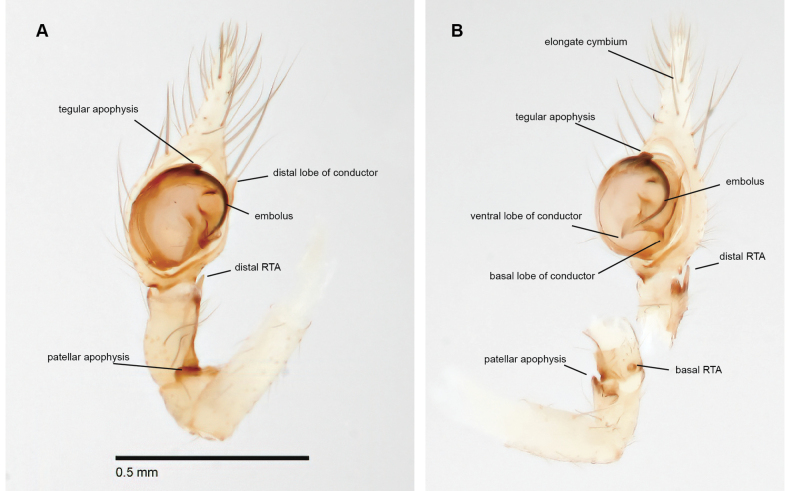
*Siskiyuarmilla* sp. nov. holotype ♂ (SDSU_G1090) palp **A** ventral view **B** lateral view. Elongate cymbium, tegular process, embolus, conductor processes, patellar apophysis, and RTA basal and distal processes labeled.

**Figure 7.**
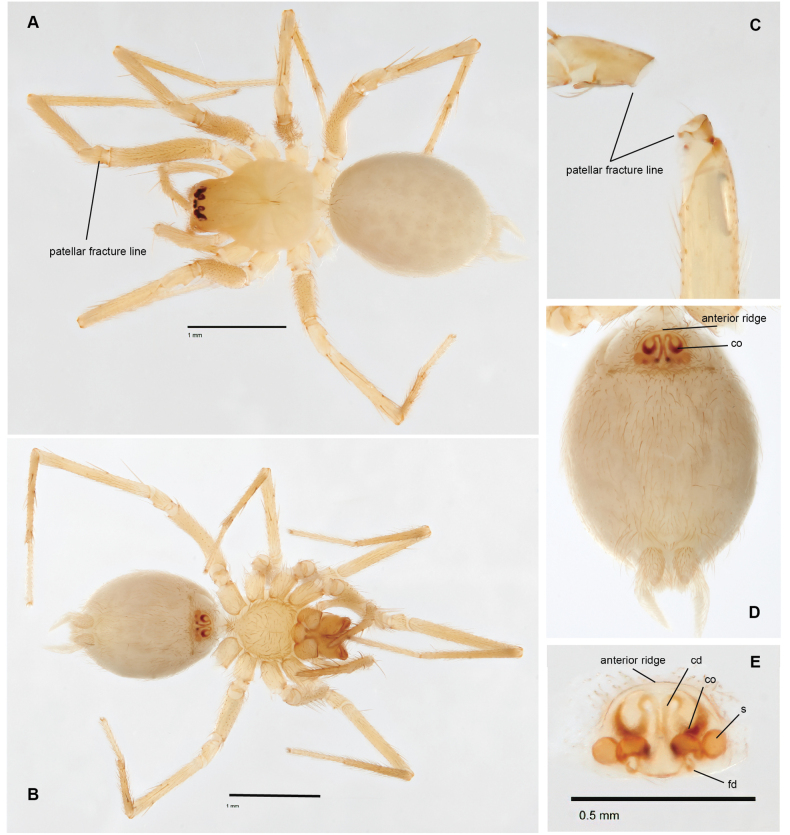
*Siskiyuarmilla* sp. nov. paratype ♀ (SDSU_G1086A) **A** dorsal view **B** ventral view **C** leg IV patellar fracture line **D** ventral epigynum **E** dorsal epigynum. Patellar fracture lines and epigynal dorsal ridge, copulatory openings (co), copulatory ducts (cd), bilobed spermathecae and fertilization ducts (fd) labeled.

With a conspicuous fracture line near the base of patellae I–IV, like close relatives *Calymmaria* and *Willisus* (Roth 1981: fig. 1; Heiss and Draney 2004: fig. 3). The male palp of *Siskiyu* gen. nov. is diagnosed from *Calymmaria* in possessing a dorsal tegular process, a thinner embolus with the distal tip projecting ventrally, associated with a trilobed conductor (Fig. 6A, B). *Calymmaria* palps lack a tegular process, have generally thicker (or sometimes forked) emboli, with conductors that lack dorsal processes (Heiss and Draney 2004: figs 6, 7). *Siskiyu* gen. nov. male palps are most like those of *Willisus* but differ in the condition of the dorsal tegular process (more robust and toothed in *Willisus*), shape of the embolus (sinuate in *Willisus*), and shapes of both the conductor and RTA lobes (Roth 1981: figs 3–5). *Siskiyu* gen. nov. female genitalia differ from *Calymmaria* in possessing subequal, bilobed spherical spermathecae (Fig. 7D, E), in contrast to the simple paired spherical spermathecae of most *Calymmaria* (Heiss and Draney 2004: figs 4, 5), and different from the distinctive *C.alleni* epigynum; Heiss and Draney 2004: figs 8, 9). *Siskiyu* gen. nov. female genitalia are distinguished from those of *Willisus* which include short copulatory ducts and oblique kidney-bean shaped spermathecae (Roth 1981: figs 6, 7).

##### Description.

Adults 2–3 mm in length. Dorsal carapace mostly bare and lacking pigmentation, longitudinal fovea ~ 2 × length of PME diameter. Eight eyes, anterior eye row slightly recurved, posterior eye row slightly procurved. AMEs smallest, ~ ¼ diameter of ALEs, separated by diameter. Lateral eyes approximately contiguous, approximately equal in size; PMEs ½ diameter of PLEs, slightly closer to PLEs. Clypeus as high as ALEs. Chelicerae straight, longer than width of cephalic region, lateral boss present; anterior margin with three teeth, posterior margin with five or six teeth. Labium nearly square, narrowing slightly anteriorly. Endites slightly convergent, ~ 2 × as long as wide, with serrula. Sternum approximately heart-shaped but with truncated anterior edge, narrowed posteriorly, ending at posterior edge of hind coxae; sparsely covered with fine hairs. Trochanters without notches. Legs with fine hairs, mostly unmarked. Leg formula 1, 4, 2, 3. Patella-tibia I 1.6–2× longer than carapace. Femurs lacking macrosetae; patellar, tibial, and metatarsal macrosetae restricted to posterior legs. Leg patellae with conspicuous cleavage plane basally at ⅕ of patella length (Fig. 7C). Tarsi and metatarsi with dorsal rows of 3–6 long trichobothria. Paired tarsal claws with ten teeth, median claw very small, semicircular hairs between claws not obvious. Abdomen with scattered simple hairs, more ventrally than dorsally. Colulus inconspicuous, with six setae. Anterior spinnerets are stout, conical, separated by basal width, distal segment hemispherical. Posterior spinnerets longer than anterior with wedge-shaped distal segments ~ 1.3× longer than length of basal segment, pointed medially. Median spinnerets lying directly posterior and approximately equal in height to anterior spinnerets, closely adjacent.

Female palp with tibial and tarsal macrosetae, clothed with fine hairs. Female genitalia with paired lateral openings, elongate copulatory ducts, bilobed spermathecae. Male palp with distal patellar apophysis; tibia with basal and distal processes. Bulb with small tegular process, slender embolus protected by beak-like ventral process of triangular conductor.

#### ﻿ Siskiyu armilla sp. nov.

Taxon classificationAnimaliaAraneaeCybaeidae

F2A4040E-5FF9-5633-B2C1-03F53A888CFE

https://zoobank.org/801103E9-B416-4D88-83D3-7E99272B927A

Figs 1 , 6 , 7 , 10

##### Material examined.

***Holotype*: – Oregon, Josephine Co.** • ♂; Hwy 46, 8.3 mi. E Cave Junction, near Nelson Creek, along Sucker Creek; 42.1643, -123.5008; 15 Aug. 2006; coll. M. Hedin, R. Keith, M. McCormack; MCH 06_115; SDSU_G1090; ***Paratypes*: – California, Siskiyou Co.** • 2♀; West Fork Beaver Creek, confluence with Little Soda Creek, Forest Road 47N01; 41.9455, -122.8334; 18 Apr. 2006; coll. M. Hedin; MCH 06_066; SDSU_G1086, SDSU_G1086A; • 3♀; Matthews Creek campground, on Salmon River; 41.1863, -123.2148; 10–12 July 2005; coll. M. Hedin; MCH 05_029.

##### Additional material.

**USA – Oregon, Josephine Co.** • ♂; Hwy 46, 8.3 mi. E Cave Junction, near Nelson Creek, along Sucker Creek; 42.1643, -123.5008; 15 Aug. 2006; coll. M. Hedin, R. Keith, M. McCormack; MCH 06_115; SDSU_G1089; – **California, Siskiyou Co.** • ♀; West Fork Beaver Creek, confluence with Little Soda Creek, Forest Road 47N01; 41.9455, -122.8334; 18 Apr. 2006; coll. M. Hedin; MCH 06_066; SDSU_G1087; • ♀; Beaver Creek Rd, near Beaver Creek campground; 41.9238, -122.8321; 18 Apr. 2006; coll. M. Hedin; MCH 06_065; SDSU_G1088; • several immatures; FR 11, along Beaver Creek, below confluence with Fishtrap Creek; 41.9426, -122.8024; 22 July 2024; coll. M. Hedin, O. Hedin; MCH 24_057; • ♀; Matthews Creek campground, on Salmon River; 41.1863, -123.2148; 10–12 July 2005; coll. M. Hedin; MCH 05_029; SDSU_G0570.

##### Etymology.

*armilla* (L., a ring, bracelet), from the conspicuous ring-like patellar fracture lines (Fig. 1C).

##### Description of ♂ holotype.

(SDSU_G1090; Fig. 6A, B) Color in alcohol pale cream to white, fangs slightly darker. Carapace essentially bare, unmarked; abdomen dusky dorsally and ventrally, slightly darker dorsal longitudinal bars posteriorly. Total length 2.3, carapace length 1.15, carapace width 0.85, cephalic region width 0.5, posterior eye row width 0.325. Eye diameters AME:ALE:PME:PLE = 0.025:0.075:0.05:0.075. Leg I article lengths (1.65, 0.5, 1.9, 1.6, 0.9 = 6.4), leg IV article lengths (1.45, 0.4, 1.5, 1.5, 0.8 = 5.6). Leg macrosetae sparse, one or two on tibia III and V (prolateral and ventral), and metatarsus III and IV (prolateral and ventral, distal cluster).

Male palp with shelf-like distal patellar apophysis. Basal retrolateral tibial apophysis (RTA) a minute spike, with sclerotized parallel weak ridges bordering a slight excavation, extending to a short and blade-like distal RTA. Bulb with a short dorsal tegular process at base of whip-like embolus which extends to ~ 3 o’clock then projects ventrally, protected by conductor. Triangular conductor with spatulate distal, and sharply pointed basal lobes. Beak-like ventral lobe of conductor enclosing embolus. Median apophysis absent. Cymbium elongate, distal projection approximately as long as bulb.

##### Description of ♀ paratype.

(SDSU_G1086A; Fig. 7A–E) Color in alcohol pale cream to orange, fangs slightly darker. Carapace essentially bare, unmarked; abdomen without markings. Total length 2.9, carapace length 1.3, carapace width 0.925, cephalic region width 0.55, posterior eye row width 0.325. Eye diameters AME:ALE:PME:PLE = 0.025:1:0.05:1. Leg I article lengths (1.57, 0.5, 1.6, 1.4, 0.9 = 5.9), leg IV article lengths (1.55, 0.4, 1.5, 1.5, 0.7 = 5.7). Leg macrosetae sparse, 1 on dorsal patella III and IV, 1–2 on tibia III and V (prolateral and ventral), and metatarsus III and IV (prolateral and ventral, distal cluster).

Epigynal plate with a semicircular ridge along the anterior edge (Fig. 7D, E). Paired lateral ear-shaped copulatory openings, sclerotized laterally and ventrally, lie ventral to this ridge. Thin copulatory ducts arise from copulatory openings and loop dorsally to meet in the midline, then extend ventrally in parallel, diverge at the bottom, then extend dorsally to connect with spermathecae. Paired spermathecae bilobed, medial lobe slightly smaller and more oval-shaped than larger and more spherical lateral lobes, connected by a short, looped duct. Fertilization ducts short and indistinct, extending ventrally from medial lobes.

##### Variation.

Female epigyna from the three geographically distant known locations (Cave Junction, Beaver Creek, Salmon River, Fig. 10) are very similar in detail.

##### Distribution and natural history.

Known from five separate locations (three in close vicinity near Beaver Creek), spanning from northern records near Cave Junction, Oregon to southern records along the Salmon River in Siskiyou County, California (Fig. 10). All collecting events are from mixed coniferous forests (including *Pseudotsugamenziesii*, *Pinus* sp., *Arbutusmenziesii*) at mid-elevations (550–800 m) in the Klamath-Siskiyou Mountains. We anticipate additional populations in intervening and neighboring locations.

Spiders found abundantly beneath rocks in shaded rock piles, often under rocks lying directly on the surface, versus deeper in rock piles. Spiders were generally found without associated webs, although some subadult specimens found along Beaver Creek at Fishtrap Creek (MCH 24_057) were found near sparse webbing (Fig. 1D). Adults have been collected in April, July, and August; only subadults were found during the July collecting event at Fishtrap Creek (MCH 24_057).

##### Comments.

Discovery of this distinctive taxon adds to our knowledge of the highly diverse flora and fauna of the Klamath-Siskiyou Mountains (Kauffmann and Garwood 2022). Other globally rare and endemic spider taxa, reflecting also the high phylogenetic diversity of the region, include *Hypochiluskastoni* Platnick, 1987, *Trogloraptormarchingtoni* Griswold, Audisio & Ledford, 2012, and *Calileptonetasylva* (Chamberlin & Ivie, 1942). Additional efforts are needed to fully understand the arthropod biodiversity of this unique region.

#### ﻿ Cybaeozyga furtiva sp. nov.

Taxon classificationAnimaliaAraneaeCybaeidae

514AFCE0-5B2A-5ABE-8B0B-D2F27531210A

https://zoobank.org/76BC699E-09AE-4ABA-B8AF-7AFCE8676EFA

Figs 8 , 9 , 10

##### Material examined.

***Holotype*: – California, Del Norte Co.** • ♀; E Crescent City, 41.8, -124.0 (GPS, ±10 km); elev. 150 m, 25–29 Jun. 2017; wet mixed forest with redwood; coll. M. Ramírez & P. Michalik; SDSU_TAC000889; ***Paratypes***: same data as holotype; • 2♀; SDSU_TAC000890; • 3♀; MACN-Ar 46970; • 2♀; MACN-Ar 38631.

##### Additional material.

– **California, Del Norte Co.** • 9♀ (together with several immatures); same data as holotype; MACN-Ar 38888; • 1♀; same data as holotype; MACN-Ar 38862; sample MJR-2128; • 1♀; same data as holotype; MACN-Ar 38880; samples MJR-1985, 1986, 1987, 1989; photos 7932–7934; • 1 ♀; same data as holotype; MACN-Ar 38948; sample MJR-2036 attachment disks; • 1♀; same data as holotype; MACN-Ar 38936; samples MJR-1985, 1987, photos 7915–7930; • 1♀; same data as holotype; MACN-Ar 38958; sample MJR-2130; • 2♀ (together with 1 immature); same data as holotype; MACN-Ar 38386; • 1♀, several immatures; southeast of Hiouchi, along South Fork Smith River, 41.76, -124.01 (GPS, ±10 km); 24 Jul. 2024; N-facing rockpile, mixed redwood forest; coll. M. Hedin & O. Hedin; – **California, Humboldt Co.** • 1♀; nr. Tish Tang Campground, SE of Hoopa; 41.01914, -123.63594; elev. 120 m; 26 Jun. 2017; mixed broadleaf forest; coll. M. Ramírez & P. Michalik; MACN-Ar 38673.

##### Etymology.

*furtiva* (L., hidden, concealed), from the rarity, and microhabitat preference, of this species.

##### Diagnosis.

The epigynum of *C.furtiva* sp. nov. differs from that of *C.heterops* Chamberlin & Ivie (1937) in possessing a slightly more sclerotized and rugous epigynal plate anterior to the epigastric furrow, with white (glandular?) material lying anterior to the spermathecae, and longer spermathecae that nearly meet at the midline.

##### Description of ♀ holotype.

**Figure 8.**
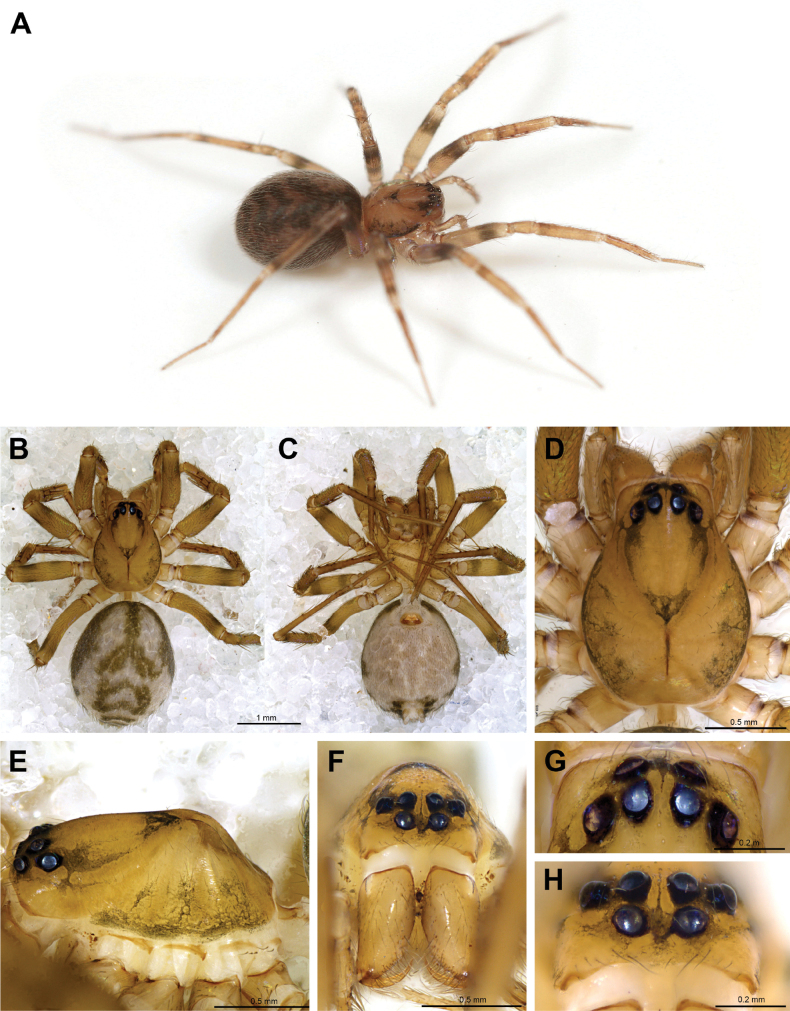
*Cybaeozygafurtiva* sp. nov. ♀ (A MACN-Ar 38880, B–H holotype) **A** live specimen MACN-Ar 38880 **B** dorsal view **C** ventral view **D** prosoma dorsal view **E** prosoma lateral view **F** prosoma anterior view **G** eyes dorsal view **H** eyes anterior view.

**Figure 9.**
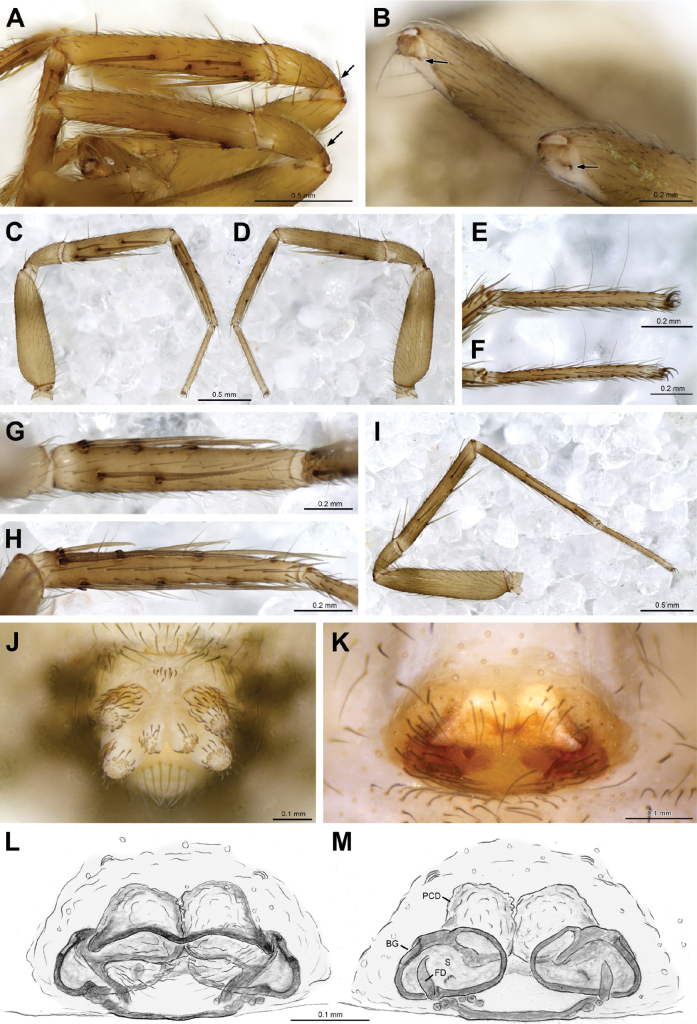
*Cybaeozygafurtiva* sp. nov. ♀ (**A, J–M** holotype **B–I** paratype MACN-Ar 46970) **A** patellae and tibiae I, arrows to fracture lines **B** patellae III–IV fractured, arrows to fracture lines **C** left leg I, prolateral view **D** same, retrolateral view **E** tarsus I prolateral view **F** tarsus IV prolateral view **G** tibia I ventral view **H** metatarsus I ventral view **I** left leg IV prolateral view **J** spinnerets ventral view **K** epigyne ventral view **L** cleared epigyne ventral view **M** cleared epigyne dorsal view. Abbreviations: BG = Bennett’s gland, FD = fertilization duct, PCD = proximal copulatory duct, S = spermatheca.

(SDSU_TAC000889; Figs 8B–H, 9A, J–M). Color in alcohol greenish brown with dark gray dorsal pattern. Carapace with dark markings extending from eyes to cephalic area, and sides of cephalic area; abdomen grayish with dark pattern dorsally, laterally and around spinnerets. Sternum unmarked. Legs darker at distal femora, basal tibiae, and basal metatarsi. Cheliceral promargin with three teeth (two basal together, one distal parted), retromargin with seven very small teeth.

Total length 3.53, carapace length 1.40, carapace width 1.05, cephalic region width 0.60, posterior eye row width 0.45. Anterior median eyes missing, represented by small dark blotches of black pigment. Eye diameters ALE:PLE:PME = 0.09:0.08:0.08. Sternum length 0.70, sternum width 0.67. Leg formula 1423. Leg I article lengths (1.27, 0.49, 1.08, 1.05, 0.75 = 4.64) leg IV article lengths (1.37, 0.45, 1.25, 2.67, 0.90 = 6.64). Abdomen 2.17 long.

Leg macrosetae (Fig. 9C–I, paratype MACN-Ar 46970) absent on femora, 1-1 on patellae (dorsal), thick on legs I–III, weaker on IV. Tibia I ventral 2-2-0, prolateral 1-1-0, dorsal 1-0 weaker; metatarsus I prolateral 1-1-1-1, ventral 2-2-2-0 not well paired. Tibia IV prolateral 1-1, ventral 1-1, retrolateral 0-1, dorsal 1-0-1; metatarsus IV prolateral 1-1, ventral 0-1-1, retrolateral 0-0-2. Patellae with basal fracture region seen as darker indentations at the sides (Fig. 9A, B).

Spinnerets short, colulus a hairy patch (Fig. 9J). Epigynal plate oval with sinuous transversal ridge and rugous median area (Fig. 9K, L). Wide proximal copulatory ducts filled with whitish material (Fig. 9K), spermathecae posterior, transversal. Copulatory openings not seen, probably in the anterior ridges and leading to the wide, soft copulatory ducts and from there to posterior spermathecae. Bennett’s gland large, on ectal side of spermatheca (Fig. 9M), fertilization ducts posteriorly placed.

##### Variation.

**Figure 10.**
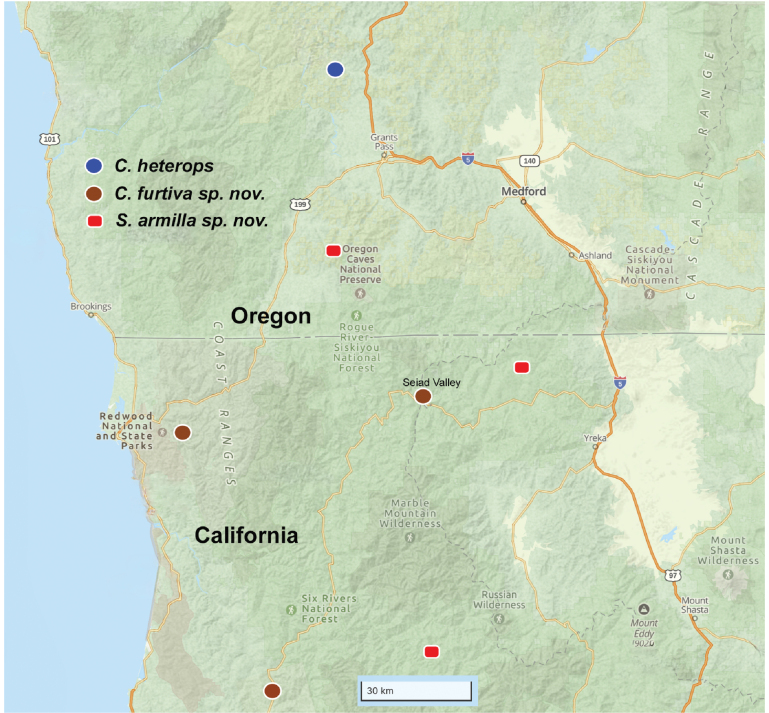
Species distributions of *Siskiyuarmilla* sp. nov. and *Cybaeozyga*, including *C.furtiva* sp. nov. and *C.heterops*. Locations approximate, see text and Suppl. material 1 for precise location data.

The black pigment replacing the missing anterior eyes is variable in location between and below the ALEs, and often asymmetrical (Fig. 8H). Epigyna from the two geographically distant locations (Fig. 10) are very similar in detail.

##### Distribution and natural history.

Confirmed specimens from Del Norte and Humboldt counties, California (Fig. 10), at lower elevations (120–150 m). Topotypic specimens were abundantly collected under rock piles or logs, and in leaf litter, in wet mixed conifer forest. Males were only represented as penultimates. Robb Bennett (pers. comm.) has examined female specimens from Siskiyou County (southeast of Seiad Valley, Fig. 10), currently housed in the American Museum of Natural History, that appear to match this species.

##### Comments.

This new species adds to the described species diversity of *Cybaeozyga*, a notoriously poorly known genus. Chamberlin and Ivie (1937) described *Cybaeozyga* and the then only known species (*C.heterops*) from a single male specimen, citing the type locality as “Grave Creek, Oregon (*near Klamath Falls*).” The authors did not include county information in their locality data. Later, Roth and Brame (1972) provided illustrations for a male *C.heterops*, matching the holotype description, and the previously undescribed female, presumably from the same location. Although precise locality data were not provided in this publication, we have seen specimens of *Cybaeozyga* from Grave Creek, Josephine County, OR collected by Roth (M. Hedin, pers. obs.). Our female specimens of *C.heterops* match closely the epigynal drawings of Roth and Brame (1972: fig. 25), and originate from Grave Creek, near confluence with Butte Creek, ~ 50 km NNW of Grants Pass, Josephine County, OR (Suppl. material 1). We view this as near the probable type locality for this species (Fig. 10); this location is approximately 200 km NW of Klamath Falls, Oregon.

Additional known, but still undescribed species of *Cybaeozyga* have been mentioned in the literature. Roth and Brame (1972) note three undescribed species from caves in northwestern California (see also Bennett et al. 2017). Overall, the distribution of *Cybaeozyga* appears to include forests and caves of the Klamath Mountains ecoregion of northwestern California and southwestern Oregon (Fig. 10).

#### ﻿ Willisus gertschi

Taxon classificationAnimaliaAraneaeCybaeidae

Roth, 1981

0FBC7844-D0EC-561E-9A2D-DE1778ED9CF3

Willisus gertschi Roth, 1981: 103, figs 1–8 (♂♀).
Willisus gertschi : Roth 1982: 7–8, figs 1, 7 (♂♀); Roth 1985: B1–6, figs 1, 7 (♂♀); Roth 1994: 53, figs 1, 7 (♂♀).

##### LSID.

[urn:lsid:nmbe.ch:spidersp:022044].

##### Additional material.

**California, San Bernardino Co.** • ♂; San Bernardino Mountains, Hwy 38, crossing of tributary of East Fork Mountain Home Creek, 34.12, -116.98; 9 September 2023; coll. M. Hedin; MCH 23_048; SDSU_G4110.

##### Variation.

We have not seen type specimens of *W.gertschi*, but descriptions and illustrations of the holotype male (Roth 1981) mostly match characters seen in the San Bernardino Mtns male specimen, including the globular conductor and robustness of the dorsal tegular process. The San Bernardino Mtns male embolus appears less sinuate than holotype illustrations (Roth 1981: figs 4, 5).

##### Comments.

Previously known only from the type locality in the San Gabriel Mountains of southern California (Mt. Baldy, Manker Flats Campground; Roth 1981). Our new records from the San Bernardino Mountains extend the distribution of this species eastward in the Los Angeles Basin. We conservatively treat these populations as conspecific until additional collections can be made. We also urge additional collecting at high elevations in neighboring mountain ranges (e.g., San Jacinto Mountains, Santa Rosa Mountains) to perhaps uncover additional records of this rare genus.

## Supplementary Material

XML Treatment for Siskiyu

XML Treatment for Siskiyu armilla

XML Treatment for Cybaeozyga furtiva

XML Treatment for Willisus gertschi

## ﻿Additional information

### Author ORCIDs

Marshal Hedin https://orcid.org/0000-0001-7683-9572

Martin J. Ramirez https://orcid.org/0000-0002-0358-0130

Rodrigo Monjaraz-Ruedas https://orcid.org/0000-0002-6462-3739

### Data availability

All of the data that support the findings of this study are available in the main text or Supplementary Information and Dryad (https://doi.org/10.5061/dryad.2v6wwpzz4) – Input matrices, analysis log files, and output tree files from phylogenetic analyses. Also, custom scripts for harvesting Sanger loci, including the reference fasta file.

## Supplementary materials

Supplementary material 1 Detailed voucher information, including voucher codes, data source, taxonomy, location information, GenBank and SRA numbers for all included taxa Data type xlsx https://binary.pensoft.net/file/1243100This dataset is made available under the Open Database License (http://opendatacommons.org/licenses/odbl/1.0/). The Open Database License (ODbL) is a license agreement intended to allow users to freely share, modify, and use this Dataset while maintaining this same freedom for others, provided that the original source and author(s) are credited.Marshal Hedin, Martin J. Ramírez, Rodrigo Monjaraz-Ruedas

Supplementary material 2 Output tree files from all phylogenetic analyses Data type pdf https://binary.pensoft.net/file/1243101This dataset is made available under the Open Database License (http://opendatacommons.org/licenses/odbl/1.0/). The Open Database License (ODbL) is a license agreement intended to allow users to freely share, modify, and use this Dataset while maintaining this same freedom for others, provided that the original source and author(s) are credited.Marshal Hedin, Martin J. Ramírez, Rodrigo Monjaraz-Ruedas
