# Supplementary material for: ﻿Phylogenomics of North American cybaeid spiders (Araneae, Cybaeidae), including the description of new taxa from the Klamath Mountains Geomorphic Province
Source: Zookeys. 2025 Feb 6;1226:47–75. doi: 10.3897/zookeys.1226.140204 (PMC11826229; doi:10.3897/zookeys.1226.140204)
Supplement: Supplementary material 2 — Output tree files from all phylogenetic analyses [file zookeys-1226-047_article-140204__-s002.pdf]

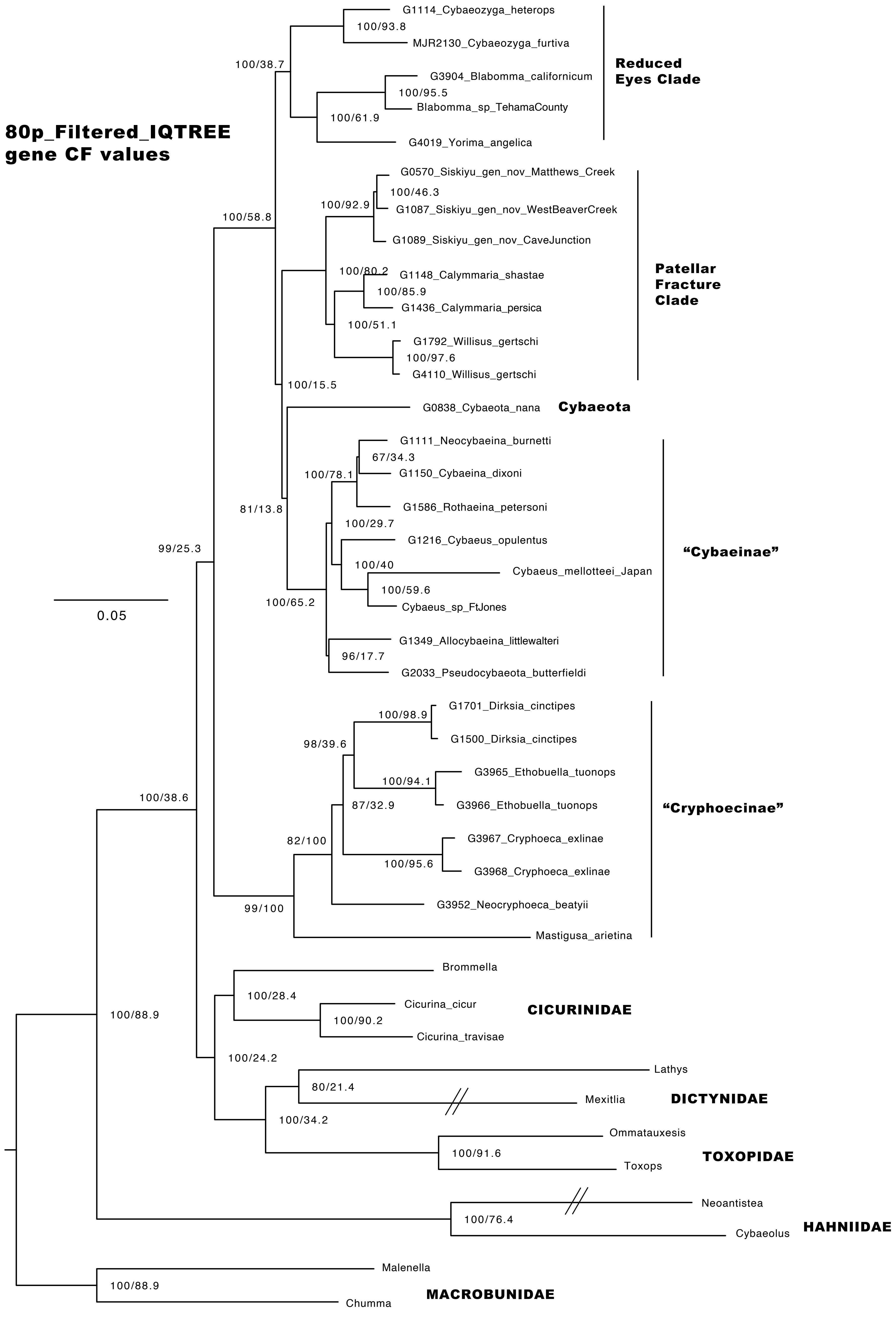
80p_Filtered_IQTREE
gene CF values
G1114_Cybaeozyga_heterops
100/93.8
MJR2130_Cybaeozyga_furtiva
100/38.7
G3904_Blabomma_californicum
100/95.5
Blabomma_sp_TehamaCounty
100/61.9
G4019_Yorima_angelica
Reduced
Eyes Clade
G0570_Siskiyu_gen_nov_Matthews_Creek
100/46.3
100/92.9
G1087_Siskiyu_gen_nov_WestBeaverCreek
100/58.8
G1089_Siskiyu_gen_nov_CaveJunction
100/80.2
G1148_Calymmaria_shastae
100/85.9
G1436_Calymmaria_persica
100/51.1
G1792_Willisus_gertschi
100/97.6
G4110_Willisus_gertschi
Patellar
Fracture
Clade
100/15.5
G0838_Cybaeota_nana
Cybaeota
G1111_Neocybaeina_burnetti
67/34.3
100/78.1
G1150_Cybaeina_dixoni
81/13.8
G1586_Rothaeina_petersoni
100/29.7
G1216_Cybaeus_opulentus
99/25.3
100/40
Cybaeus_mellotteei_Japan
100/59.6
Cybaeus_sp_FtJones
100/65.2
G1349_Allocybaeina_littlewalteri
96/17.7
G2033_Pseudocybaeota_butterfieldi
"Cybaeinae"
0.05
100/98.9
G1701_Dirksia_cinctipes
98/39.6
G1500_Dirksia_cinctipes
G3965_Ethobuella_tuonops
100/94.1
100/38.6
87/32.9
G3966_Ethobuella_tuonops
"Cryphoecinae"
82/100
G3967_Cryphoeca_exlinae
100/95.6
G3968_Cryphoeca_exlinae
99/100
G3952_Neocryphoeca_beatyii
Mastigusa_arietina
Brommella
100/28.4
Cicurina_cicur
CICURINIDAE
100/88.9
100/90.2
Cicurina_travisae
100/24.2
Lathys
80/21.4
Mexitlia
DICTYNIDAE
100/34.2
Ommatauxesis
100/91.6
TOXOPIDAE
Toxops
Neoantistea
100/76.4
Cybaeolus
HAHNIIDAE
Malenella
100/88.9
Chumma
MACROBUNIDAE

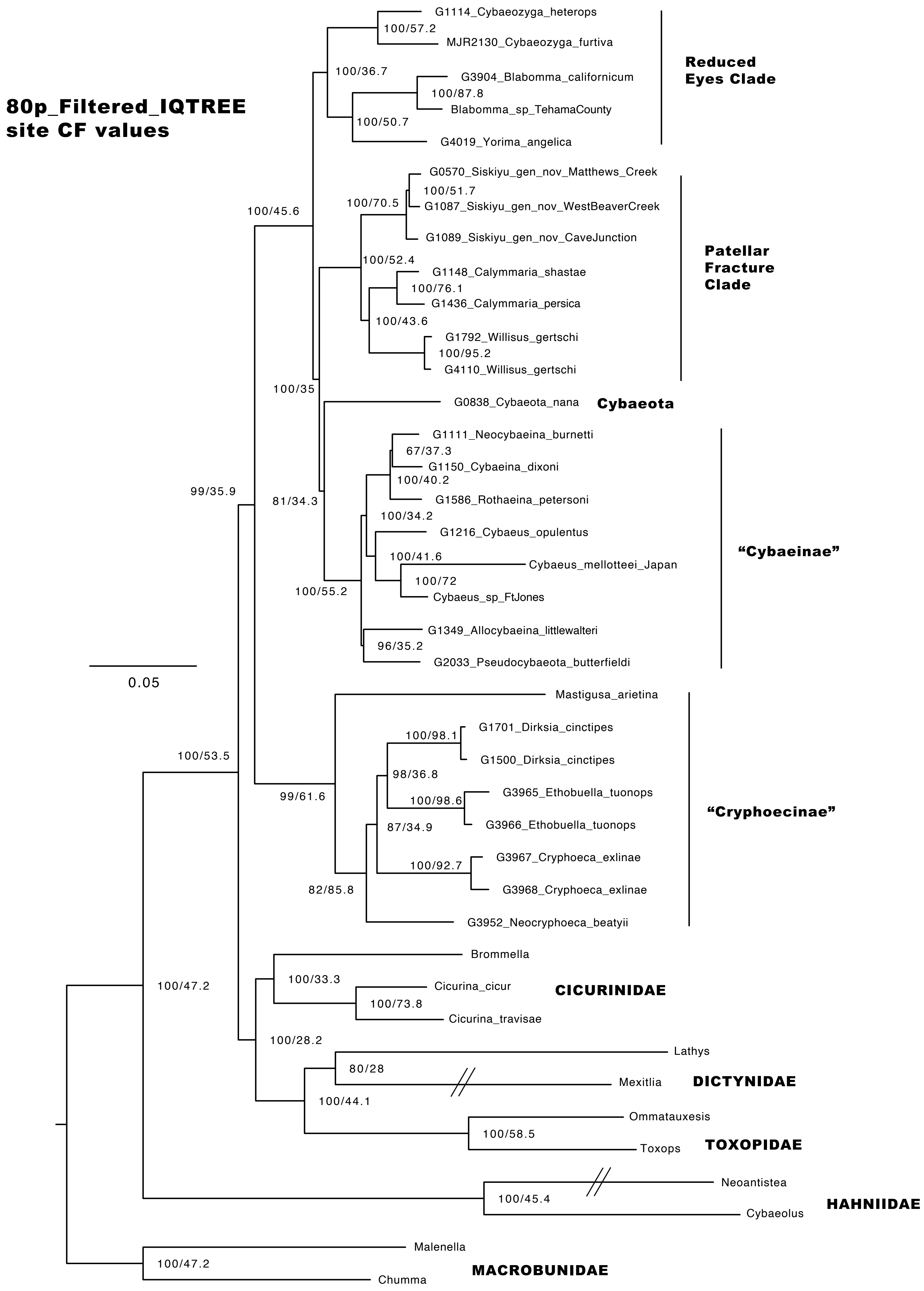
80p_Filtered_IQTREE site CF values
G1114_Cybaeozyga_heterops
100/57.2
MJR2130_Cybaeozyga_furtiva
100/36.7
G3904_Blabomma_californicum
100/87.8
Blabomma_sp_TehamaCounty
100/50.7
G4019_Yorima_angelica
Reduced Eyes Clade
G0570_Siskiyu_gen_nov_Matthews_Creek
100/51.7
100/70.5
G1087_Siskiyu_gen_nov_WestBeaverCreek
100/45.6
G1089_Siskiyu_gen_nov_CaveJunction
100/52.4
G1148_Calymmaria_shastae
100/76.1
G1436_Calymmaria_persica
100/43.6
G1792_Willisus_gertschi
100/95.2
G4110_Willisus_gertschi
Patellar Fracture Clade
100/35
G0838_Cybaeota_nana
Cybaeota
G1111_Neocybaeina_burnetti
67/37.3
G1150_Cybaeina_dixoni
100/40.2
99/35.9
81/34.3
G1586_Rothaeina_petersoni
100/34.2
G1216_Cybaeus_opulentus
100/41.6
Cybaeus_mellotteei_Japan
100/72
Cybaeus_sp_FtJones
100/55.2
G1349_Allocybaeina_littlewalteri
96/35.2
G2033_Pseudocybaeota_butterfieldi
"Cybaeinae"
0.05
Mastigusa_arietina
G1701_Dirksia_cinctipes
100/98.1
G1500_Dirksia_cinctipes
100/53.5
98/36.8
G3965_Ethobuella_tuonops
99/61.6
100/98.6
G3966_Ethobuella_tuonops
87/34.9
G3967_Cryphoeca_exlinae
100/92.7
82/85.8
G3968_Cryphoeca_exlinae
G3952_Neocryphoeca_beatyii
"Cryphoecinae"
Brommella
100/33.3
Cicurina_cicur
100/47.2
100/73.8
CICURINIDAE
Cicurina_travisae
100/28.2
Lathys
80/28
Mexitlia
DICTYNIDAE
100/44.1
Ommatauxesis
100/58.5
Toxops
TOXOPIDAE
Neoantistea
100/45.4
Cybaeolus
HAHNIIDAE
Malenella
100/47.2
Chumma
MACROBUNIDAE

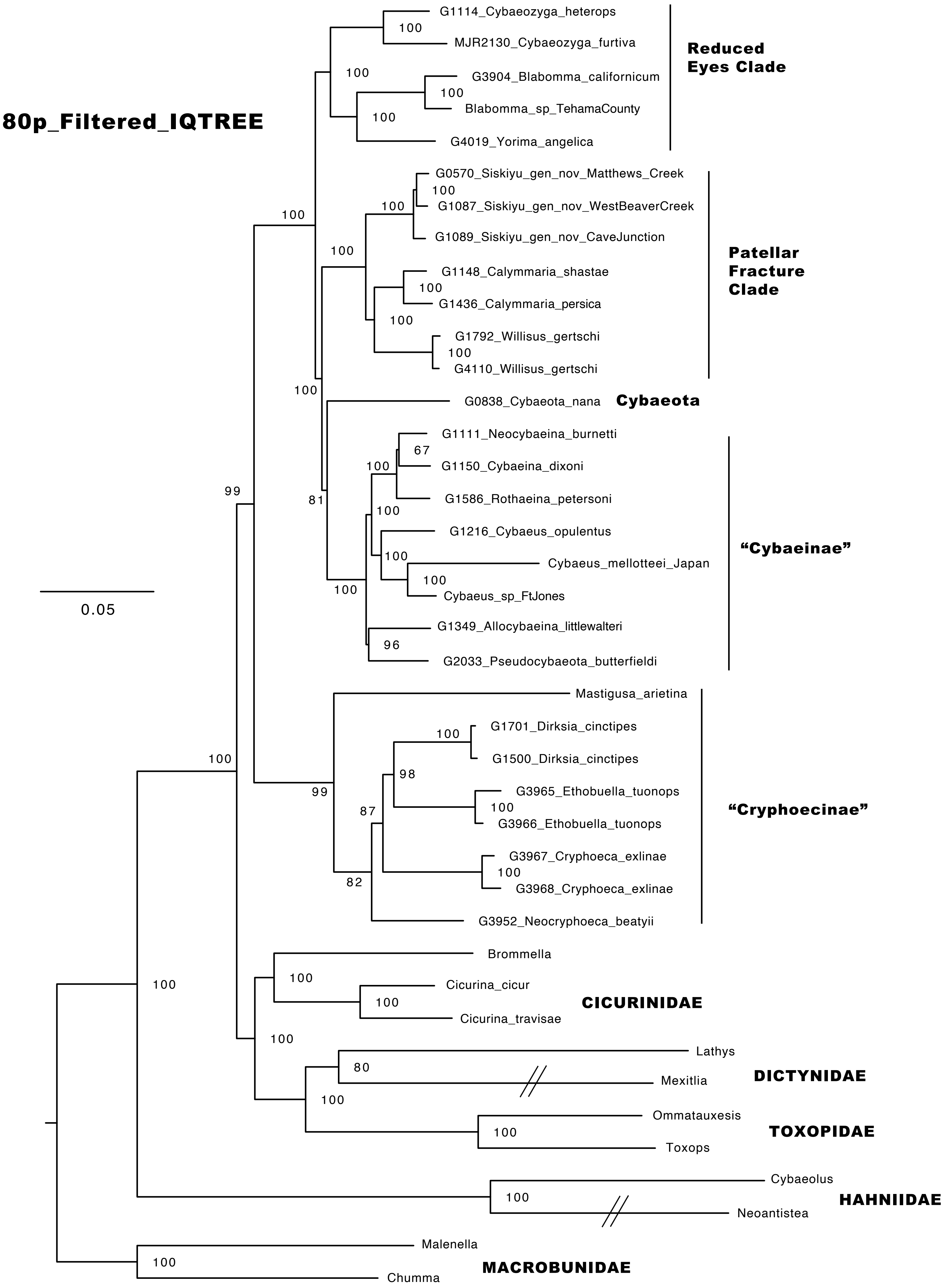
80p_Filtered_IQTREE
0.05
G1114_Cybaeozyga_heterops
MJR2130_Cybaeozyga_furtiva
G3904_Blabomma_californicum
Blabomma_sp_TehamaCounty
G4019_Yorima_angelica
Reduced Eyes Clade
G0570_Siskiyu_gen_nov_Matthews_Creek
G1087_Siskiyu_gen_nov_WestBeaverCreek
G1089_Siskiyu_gen_nov_CaveJunction
G1148_Calymmaria_shastae
G1436_Calymmaria_persica
G1792_Willisus_gertschi
G4110_Willisus_gertschi
Patellar Fracture Clade
G0838_Cybaeota_nana
Cybaeota
G1111_Neocybaeina_burnetti
G1150_Cybaeina_dixoni
G1586_Rothaeina_petersoni
G1216_Cybaeus_opulentus
Cybaeus_mellotteei_Japan
Cybaeus_sp_FtJones
G1349_Allocybaeina_littlewalteri
G2033_Pseudocybaeota_butterfieldi
“Cybaeinae”
Mastigusa_arietina
G1701_Dirksia_cinctipes
G1500_Dirksia_cinctipes
G3965_Ethobuella_tuonops
G3966_Ethobuella_tuonops
G3967_Cryphoeca_exlinae
G3968_Cryphoeca_exlinae
G3952_Neocryphoeca_beatyii
“Cryphoecinae”
Brommella
Cicurina_cicur
Cicurina_travisae
CICURINIDAE
Lathys
Mexitlia
DICTYNIDAE
Ommatauxesis
Toxops
TOXOPIDAE
Cybaeolus
Neoantistea
HAHNIIDAE
Malenella
Chumma
MACROBUNIDAE

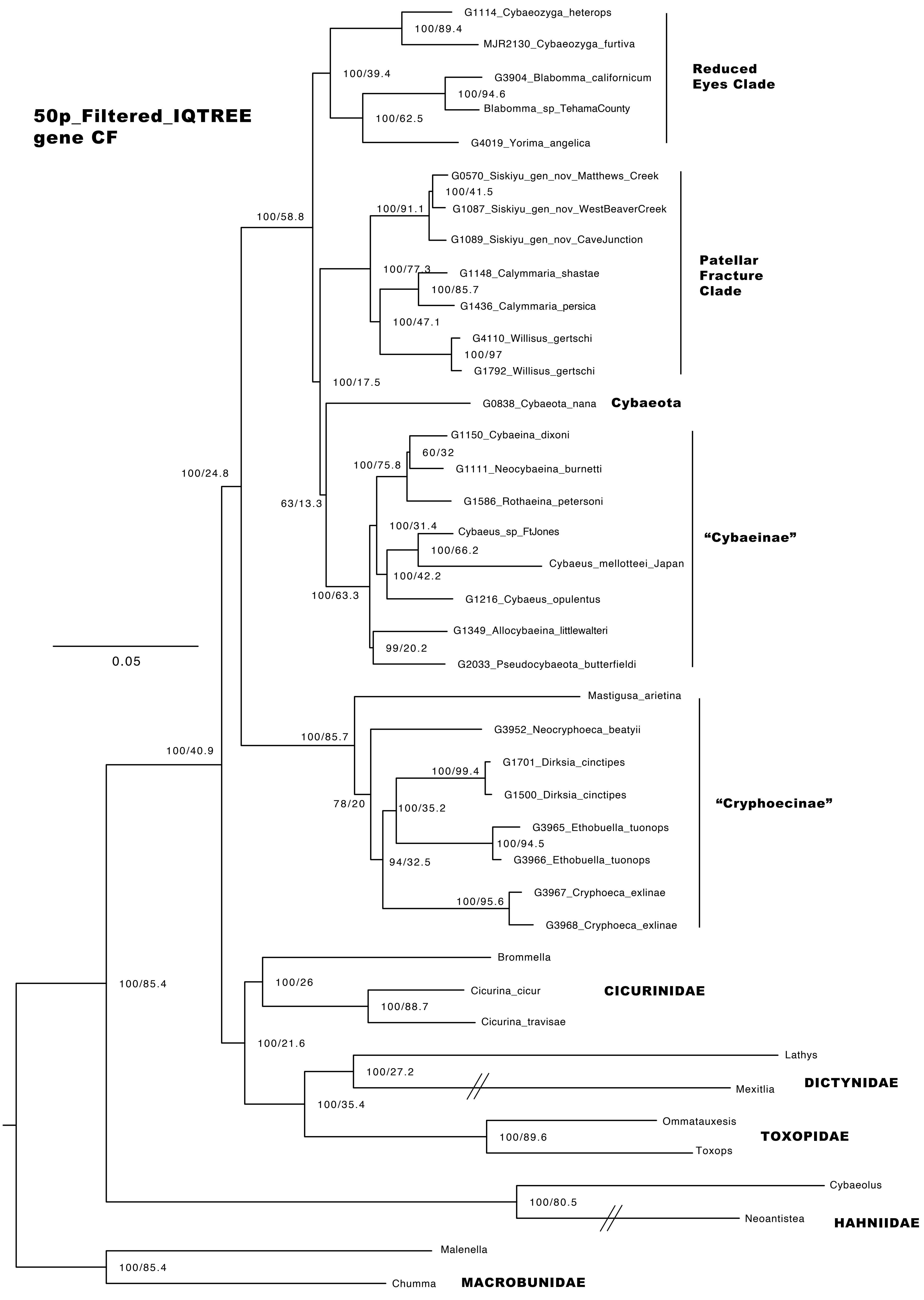
50p_Filtered_IQTREE
gene CF
G1114_Cybaeozyga_heterops
100/89.4
MJR2130_Cybaeozyga_furtiva
100/39.4
G3904_Blabomma_californicum
100/94.6
Blabomma_sp_TehamaCounty
100/62.5
G4019_Yorima_angelica
Reduced
Eyes Clade
G0570_Siskiyu_gen_nov_Matthews_Creek
100/41.5
G1087_Siskiyu_gen_nov_WestBeaverCreek
100/91.1
G1089_Siskiyu_gen_nov_CaveJunction
100/58.8
100/77.3
G1148_Calymmaria_shastae
100/85.7
G1436_Calymmaria_persica
100/47.1
G4110_Willisus_gertschi
100/97
G1792_Willisus_gertschi
Patellar
Fracture
Clade
100/17.5
G0838_Cybaeota_nana
Cybaeota
G1150_Cybaeina_dixoni
60/32
G1111_Neocybaeina_burnetti
100/75.8
100/24.8
G1586_Rothaeina_petersoni
63/13.3
100/31.4
Cybaeus_sp_FtJones
100/66.2
Cybaeus_mellotteei_Japan
100/42.2
G1216_Cybaeus_opulentus
100/63.3
“Cybaeinae”
G1349_Allocybaeina_littlewalteri
99/20.2
G2033_Pseudocybaeota_butterfieldi
0.05
Mastigusa_arietina
G3952_Neocryphoeca_beatyii
100/85.7
100/40.9
G1701_Dirksia_cinctipes
100/99.4
G1500_Dirksia_cinctipes
78/20
100/35.2
G3965_Ethobuella_tuonops
100/94.5
G3966_Ethobuella_tuonops
94/32.5
“Cryphoecinae”
G3967_Cryphoeca_exlinae
100/95.6
G3968_Cryphoeca_exlinae
Brommella
100/26
Cicurina_cicur
CICURINIDAE
100/85.4
100/88.7
Cicurina_travisae
100/21.6
Lathys
100/27.2
Mexitlia
DICTYNIDAE
100/35.4
Ommatauxesis
100/89.6
TOXOPIDAE
Toxops
Cybaeolus
100/80.5
Neoantistea
HAHNIIDAE
Malenella
100/85.4
Chumma
MACROBUNIDAE

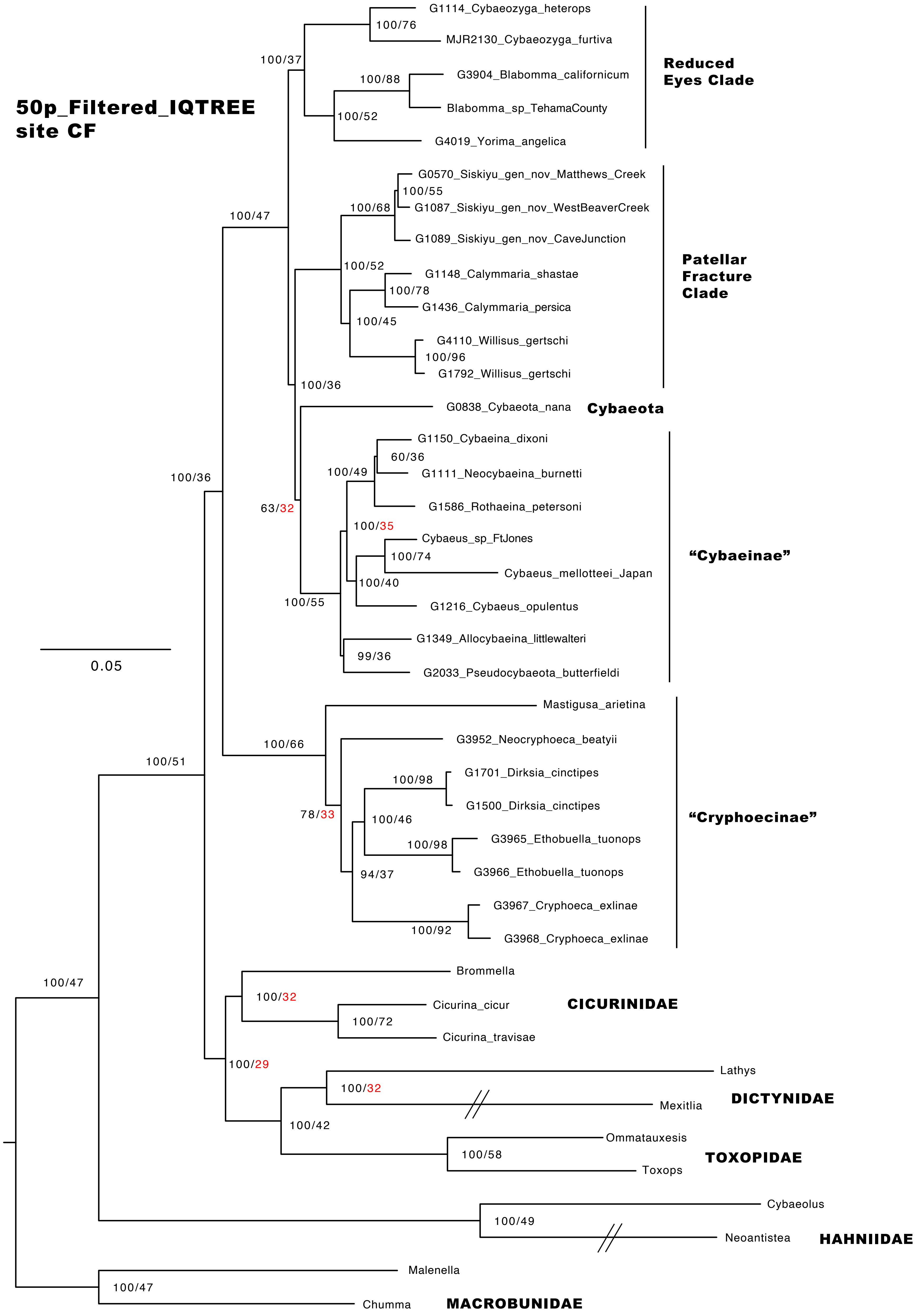
50p_Filtered_IQTREE site CF
G1114_Cybaeozyga_heterops
100/76
MJR2130_Cybaeozyga_furtiva
100/37
100/88
G3904_Blabomma_californicum
Blabomma_sp_TehamaCounty
100/52
G4019_Yorima_angelica
Reduced Eyes Clade
G0570_Siskiyu_gen_nov_Matthews_Creek
100/55
100/68
G1087_Siskiyu_gen_nov_WestBeaverCreek
100/47
G1089_Siskiyu_gen_nov_CaveJunction
100/52
G1148_Calymmaria_shastae
100/78
G1436_Calymmaria_persica
100/45
G4110_Willisus_gertschi
100/96
G1792_Willisus_gertschi
Patellar Fracture Clade
100/36
G0838_Cybaeota_nana
Cybaeota
G1150_Cybaeina_dixoni
60/36
100/49
G1111_Neocybaeina_burnetti
100/36
G1586_Rothaeina_petersoni
63/32
100/35
Cybaeus_sp_FtJones
100/74
Cybaeus_mellotteei_Japan
100/40
G1216_Cybaeus_opulentus
100/55
G1349_Allocybaeina_littlewalteri
99/36
G2033_Pseudocybaeota_butterfieldi
"Cybaeinae"
0.05
Mastigusa_arietina
100/66
G3952_Neocryphoeca_beatyii
100/51
100/98
G1701_Dirksia_cinctipes
G1500_Dirksia_cinctipes
78/33
100/46
100/98
G3965_Ethobuella_tuonops
94/37
G3966_Ethobuella_tuonops
G3967_Cryphoeca_exlinae
100/92
G3968_Cryphoeca_exlinae
"Cryphoecinae"
Brommella
100/47
100/32
Cicurina_cicur
CICURINIDAE
100/72
Cicurina_travisae
100/29
Lathys
100/32
Mexitlia
DICTYNIDAE
100/42
Ommatauxesis
100/58
TOXOPIDAE
Toxops
Cybaeolus
100/49
Neoantistea
HAHNIIDAE
Malenella
100/47
Chumma
MACROBUNIDAE

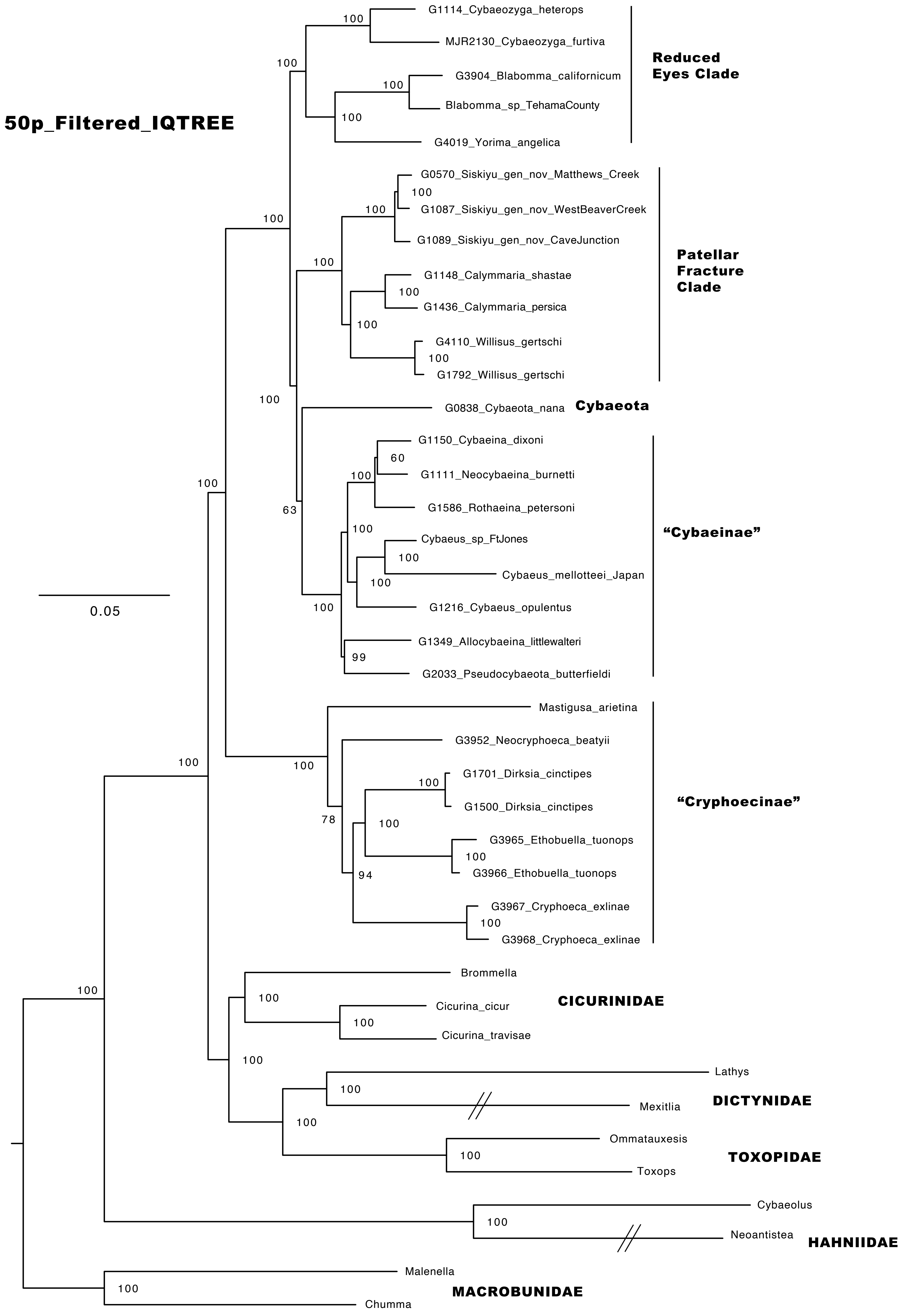

50p_Filtered_IQTREE
0.05
G1114_Cybaeozyga_heterops
MJR2130_Cybaeozyga_furtiva
G3904_Blabomma_californicum
Blabomma_sp_TehamaCounty
G4019_Yorima_angelica
Reduced Eyes Clade
G0570_Siskiyu_gen_nov_Matthews_Creek
G1087_Siskiyu_gen_nov_WestBeaverCreek
G1089_Siskiyu_gen_nov_CaveJunction
G1148_Calymmaria_shastae
G1436_Calymmaria_persica
G4110_Willisus_gertschi
G1792_Willisus_gertschi
Patellar Fracture Clade
G0838_Cybaeota_nana
Cybaeota
G1150_Cybaeina_dixoni
G1111_Neocybaeina_burnetti
G1586_Rothaeina_petersoni
Cybaeus_sp_FtJones
Cybaeus_mellotteei_Japan
G1216_Cybaeus_opulentus
G1349_Allocybaeina_littlewalteri
G2033_Pseudocybaeota_butterfieldi
“Cybaeinae”
Mastigusa_arietina
G3952_Neocryphoeca_beatyii
G1701_Dirksia_cinctipes
G1500_Dirksia_cinctipes
G3965_Ethobuella_tuonops
G3966_Ethobuella_tuonops
G3967_Cryphoeca_exlinae
G3968_Cryphoeca_exlinae
“Cryphoecinae”
Brommella
Cicurina_cicur
Cicurina_travisae
CICURINIDAE
Lathys
Mexitlia
DICTYNIDAE
Ommatauxesis
Toxops
TOXOPIDAE
Cybaeolus
Neoantistea
HAHNIIDAE
Malenella
Chumma
MACROBUNIDAE
100
60
63
78
94
99

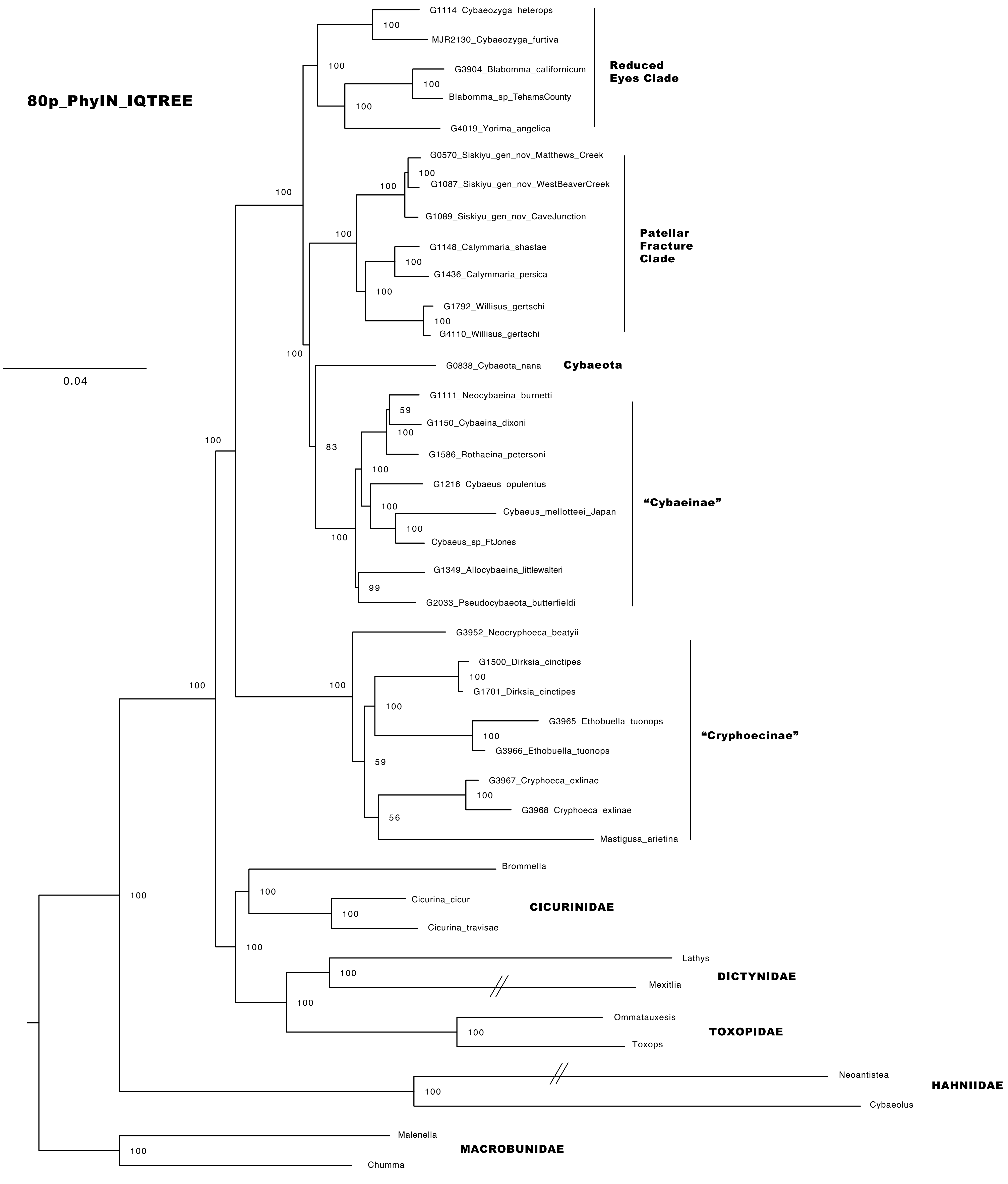
80p_PhyIN_IQTREE
0.04
G1114_Cybaeozyga_heterops
MJR2130_Cybaeozyga_furtiva
G3904_Blabomma_californicum
Blabomma_sp_TehamaCounty
G4019_Yorima_angelica
Reduced Eyes Clade
G0570_Siskiyu_gen_nov_Matthews_Creek
G1087_Siskiyu_gen_nov_WestBeaverCreek
G1089_Siskiyu_gen_nov_CaveJunction
G1148_Calymmaria_shastae
G1436_Calymmaria_persica
G1792_Willisus_gertschi
G4110_Willisus_gertschi
Patellar Fracture Clade
G0838_Cybaeota_nana
Cybaeota
G1111_Neocybaeina_burnetti
G1150_Cybaeina_dixoni
G1586_Rothaeina_petersoni
G1216_Cybaeus_opulentus
Cybaeus_mellotteei_Japan
Cybaeus_sp_FtJones
G1349_Allocybaeina_littlewalteri
G2033_Pseudocybaeota_butterfieldi
“Cybaeinae”
G3952_Neocryphoeca_beatyii
G1500_Dirksia_cinctipes
G1701_Dirksia_cinctipes
G3965_Ethobuella_tuonops
G3966_Ethobuella_tuonops
G3967_Cryphoeca_exlinae
G3968_Cryphoeca_exlinae
Mastigusa_arietina
“Cryphoecinae”
Brommella
Cicurina_cicur
Cicurina_travisae
CICURINIDAE
Lathys
Mexitlia
DICTYNIDAE
Ommatauxesis
Toxops
TOXOPIDAE
Neoantistea
Cybaeolus
HAHNIIDAE
Malenella
Chumma
MACROBUNIDAE

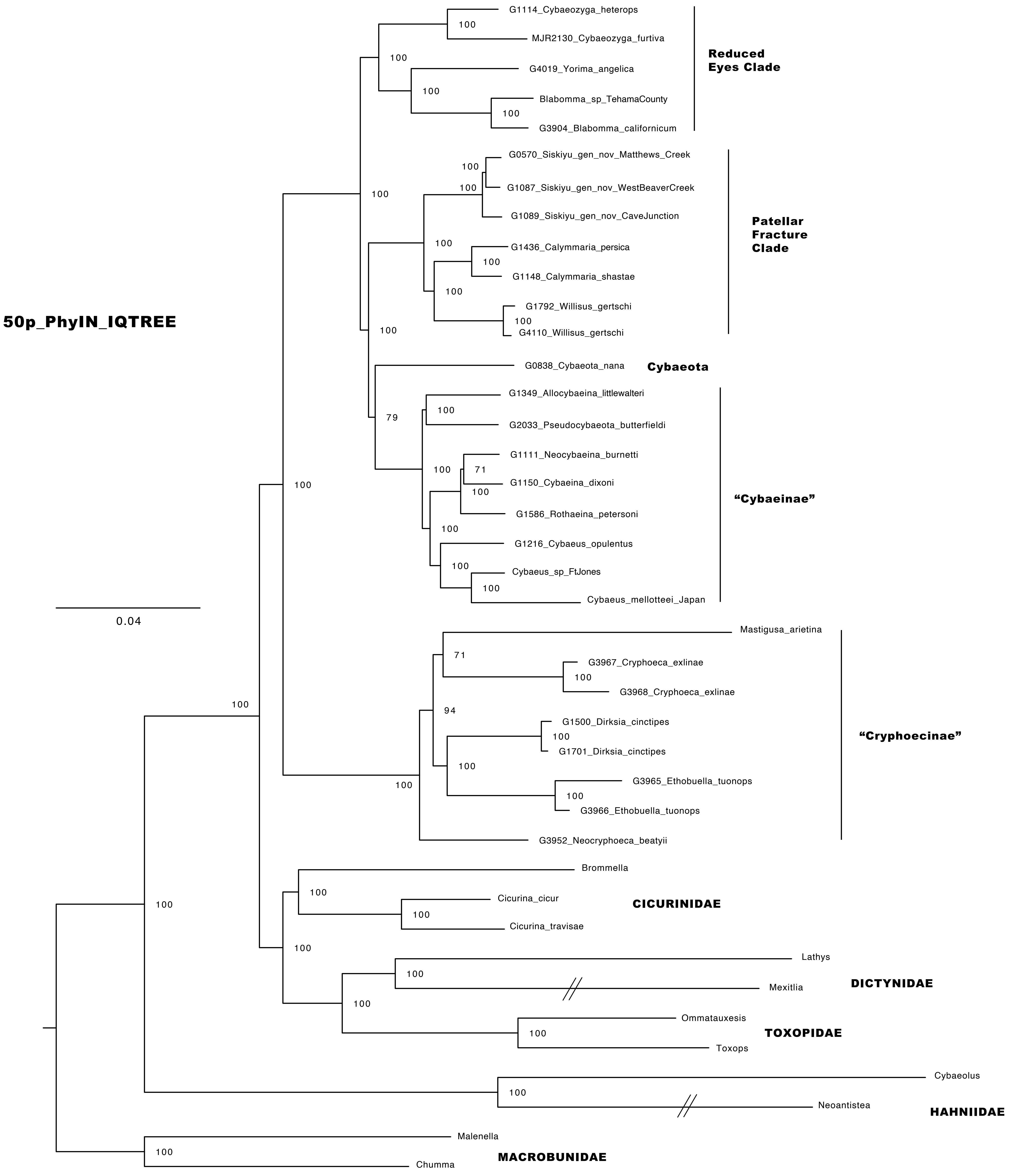

50p_PhyIN_IQTREE
0.04
G1114_Cybaeozyga_heterops
MJR2130_Cybaeozyga_furtiva
G4019_Yorima_angelica
Blabomma_sp_TehamaCounty
G3904_Blabomma_californicum
Reduced Eyes Clade
G0570_Siskiyu_gen_nov_Matthews_Creek
G1087_Siskiyu_gen_nov_WestBeaverCreek
G1089_Siskiyu_gen_nov_CaveJunction
G1436_Calymmaria_persica
G1148_Calymmaria_shastae
G1792_Willisus_gertschi
G4110_Willisus_gertschi
Patellar Fracture Clade
G0838_Cybaeota_nana
Cybaeota
G1349_Allocybaeina_littlewalteri
G2033_Pseudocybaeota_butterfieldi
G1111_Neocybaeina_burnetti
G1150_Cybaeina_dixoni
G1586_Rothaeina_petersoni
G1216_Cybaeus_opulentus
Cybaeus_sp_FtJones
Cybaeus_mellotteei_Japan
"Cybaeinae"
Mastigusa_arietina
G3967_Cryphoeca_exlinae
G3968_Cryphoeca_exlinae
G1500_Dirksia_cinctipes
G1701_Dirksia_cinctipes
G3965_Ethobuella_tuonops
G3966_Ethobuella_tuonops
G3952_Neocryphoeca_beatyii
"Cryphoecinae"
Brommella
Cicurina_cicur
Cicurina_travisae
CICURINIDAE
Lathys
Mexitlia
DICTYNIDAE
Ommatauxesis
Toxops
TOXOPIDAE
Cybaeolus
Neoantistea
HAHNIIDAE
Malenella
Chumma
MACROBUNIDAE

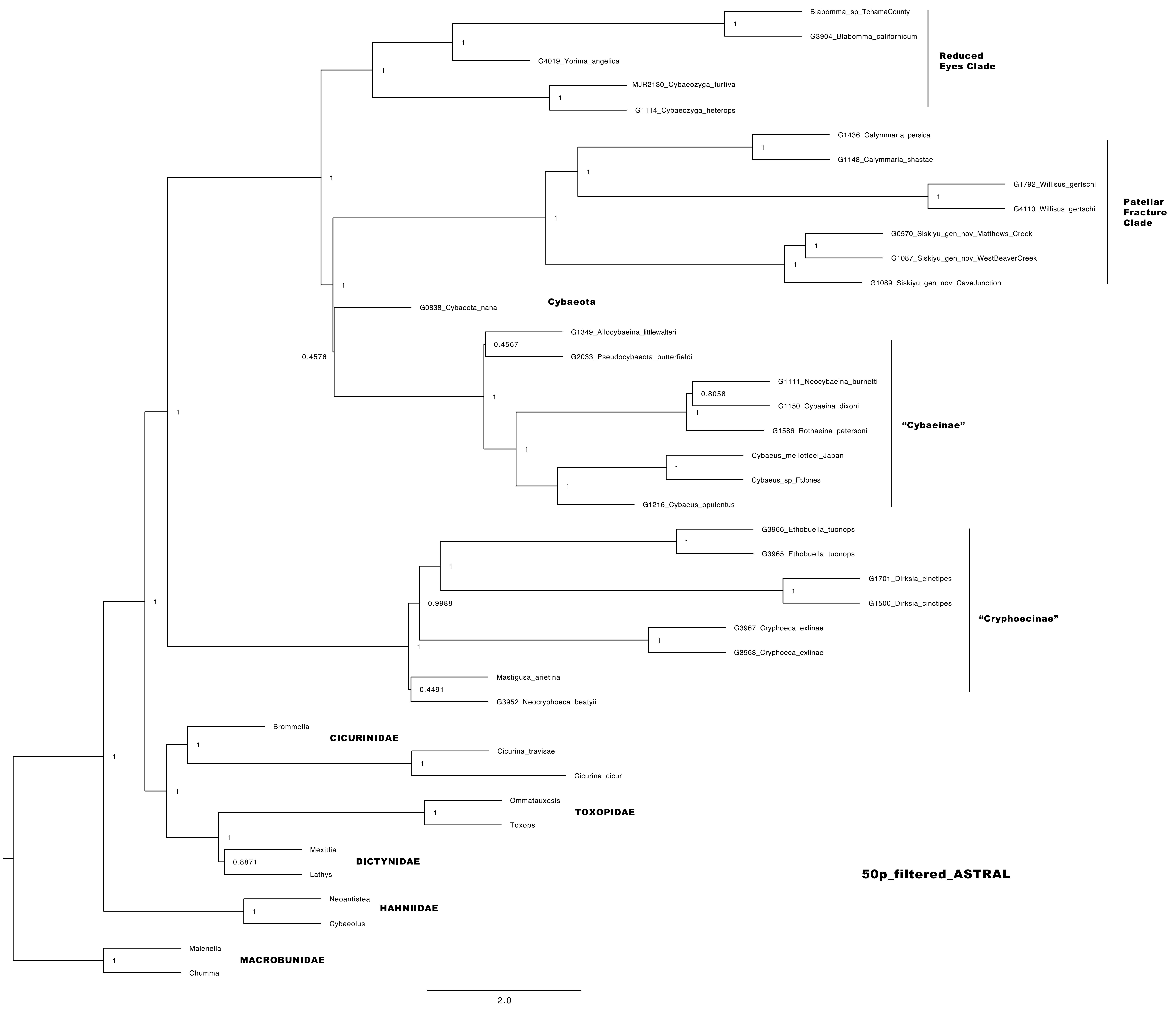
Blabomma_sp_TehamaCounty
G3904_Blabomma_californicum
G4019_Yorima_angelica
MJR2130_Cybaeozyga_furtiva
G1114_Cybaeozyga_heterops
Reduced Eyes Clade
G1436_Calymmaria_persica
G1148_Calymmaria_shastae
G1792_Willisus_gertschi
G4110_Willisus_gertschi
G0570_Siskiyu_gen_nov_Matthews_Creek
G1087_Siskiyu_gen_nov_WestBeaverCreek
G1089_Siskiyu_gen_nov_CaveJunction
Patellar Fracture Clade
G0838_Cybaeota_nana
Cybaeota
G1349_Allocybaeina_littlewalteri
G2033_Pseudocybaeota_butterfieldi
G1111_Neocybaeina_burnetti
G1150_Cybaeina_dixoni
G1586_Rothaeina_petersoni
Cybaeus_mellotteei_Japan
Cybaeus_sp_FtJones
G1216_Cybaeus_opulentus
"Cybaeinae"
G3966_Ethobuella_tuonops
G3965_Ethobuella_tuonops
G1701_Dirksia_cinctipes
G1500_Dirksia_cinctipes
G3967_Cryphoeca_exlinae
G3968_Cryphoeca_exlinae
Mastigusa_arietina
G3952_Neocryphoeca_beatyii
"Cryphoecinae"
Brommella
CICURINIDAE
Cicurina_travisae
Cicurina_cicur
Ommatauxesis
Toxops
TOXOPIDAE
Mexitlia
Lathys
DICTYNIDAE
Neoantistea
Cybaeolus
HAHNIIDAE
Malenella
Chumma
MACROBUNIDAE
0.4576
0.4567
0.8058
0.9988
0.4491
0.8871
2.0
50p_filtered_ASTRAL

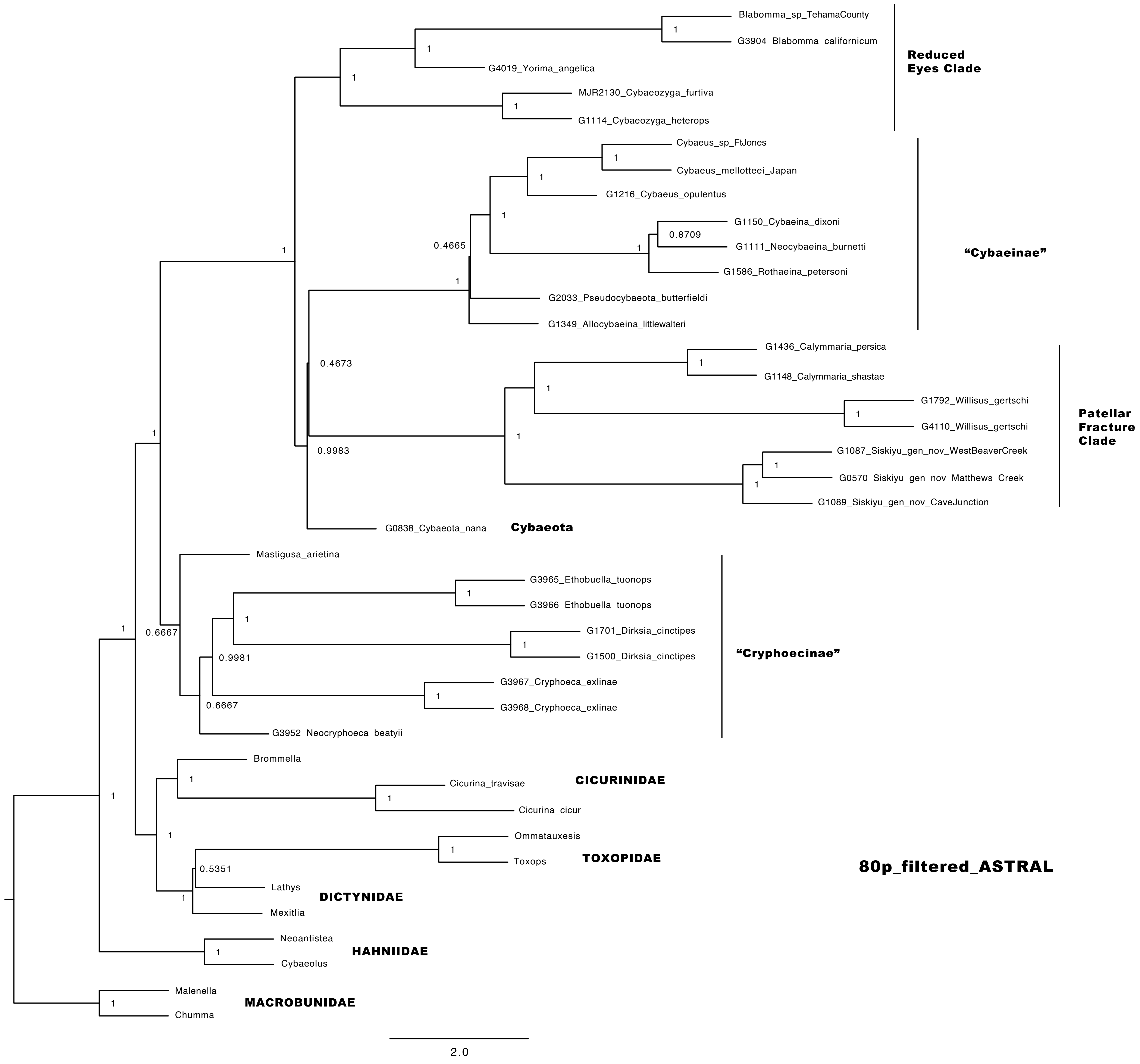
Blabomma_sp_TehamaCounty
G3904_Blabomma_californicum
G4019_Yorima_angelica
MJR2130_Cybaeozyga_furtiva
G1114_Cybaeozyga_heterops
Reduced Eyes Clade
Cybaeus_sp_FtJones
Cybaeus_mellotteei_Japan
G1216_Cybaeus_opulentus
G1150_Cybaeina_dixoni
G1111_Neocybaeina_burnetti
G1586_Rothaeina_petersoni
G2033_Pseudocybaeota_butterfieldi
G1349_Allocybaeina_littlewalteri
"Cybaeinae"
G1436_Calymmaria_persica
G1148_Calymmaria_shastae
G1792_Willisus_gertschi
G4110_Willisus_gertschi
G1087_Siskiyu_gen_nov_WestBeaverCreek
G0570_Siskiyu_gen_nov_Matthews_Creek
G1089_Siskiyu_gen_nov_CaveJunction
Patellar Fracture Clade
G0838_Cybaeota_nana
Cybaeota
Mastigusa_arietina
G3965_Ethobuella_tuonops
G3966_Ethobuella_tuonops
G1701_Dirksia_cinctipes
G1500_Dirksia_cinctipes
G3967_Cryphoeca_exlinae
G3968_Cryphoeca_exlinae
G3952_Neocryphoeca_beatyii
"Cryphoecinae"
Brommella
Cicurina_travisae
Cicurina_cicur
CICURINIDAE
Ommatauxesis
Toxops
TOXOPIDAE
Lathys
Mexitlia
DICTYNIDAE
Neoantistea
Cybaeolus
HAHNIIDAE
Malenella
Chumma
MACROBUNIDAE
0.4665
0.8709
0.4673
0.9983
0.6667
0.9981
0.6667
0.5351
2.0
80p_filtered_ASTRAL

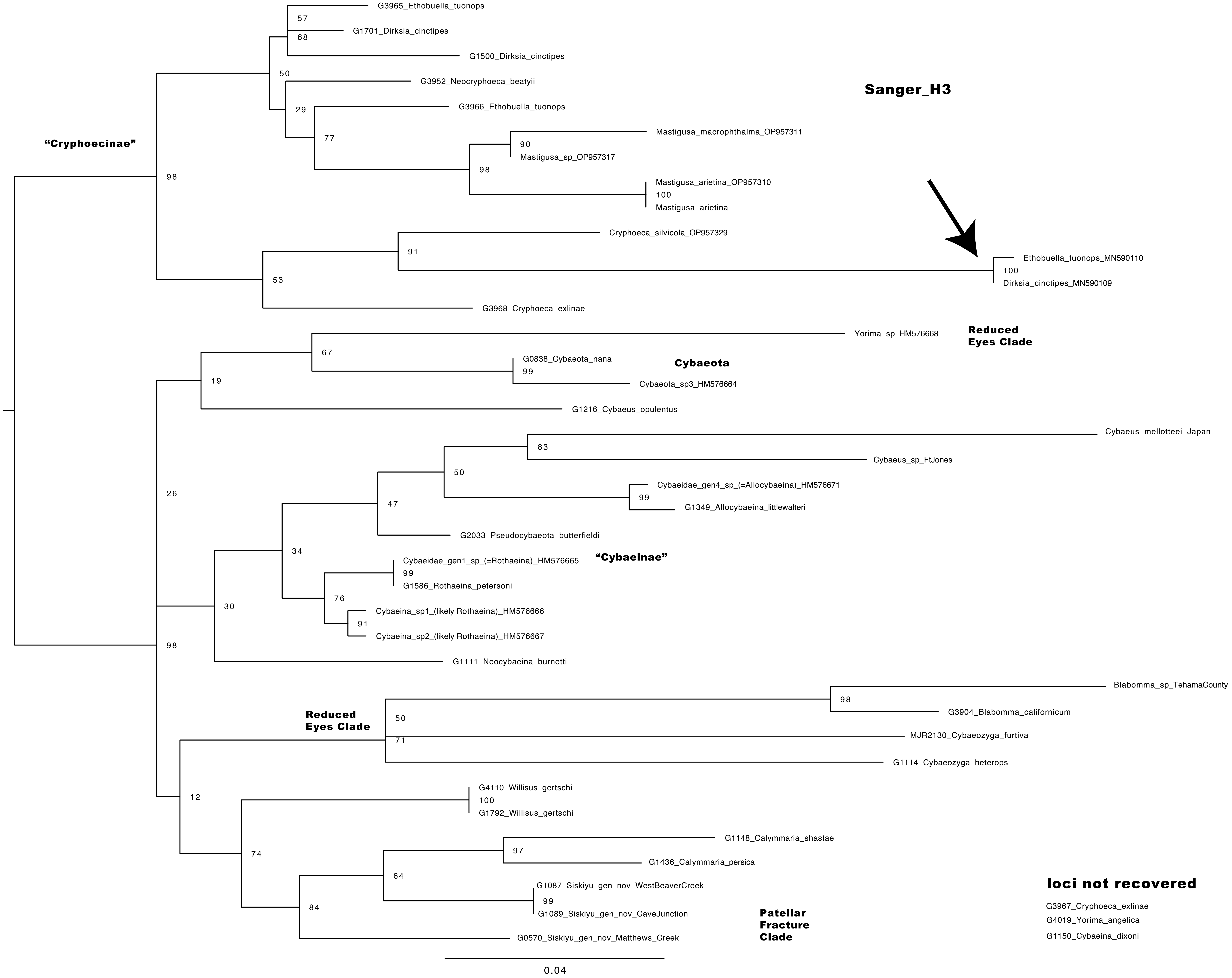

Sanger_H3
"Cryphoecinae"
G3965_Ethobuella_tuonops
57
G1701_Dirksia_cinctipes
68
G1500_Dirksia_cinctipes
50
G3952_Neocryphoeca_beatyii
29
G3966_Ethobuella_tuonops
77
Mastigusa_macrophthalma_OP957311
90
Mastigusa_sp_OP957317
98
Mastigusa_arietina_OP957310
100
Mastigusa_arietina
98
Cryphoeca_silvicola_OP957329
91
Ethobuella_tuonops_MN590110
100
Dirksia_cinctipes_MN590109
53
G3968_Cryphoeca_exlinae
Yorima_sp_HM576668
Reduced Eyes Clade
67
G0838_Cybaeota_nana
Cybaeota
99
Cybaeota_sp3_HM576664
19
G1216_Cybaeus_opulentus
26
Cybaeus_mellotteei_Japan
83
Cybaeus_sp_FtJones
50
Cybaeidae_gen4_sp_(=Allocybaeina)_HM576671
99
G1349_Allocybaeina_littlewalteri
47
G2033_Pseudocybaeota_butterfieldi
34
Cybaeidae_gen1_sp_(=Rothaeina)_HM576665
"Cybaeinae"
99
G1586_Rothaeina_petersoni
76
Cybaeina_sp1_(likely Rothaeina)_HM576666
91
Cybaeina_sp2_(likely Rothaeina)_HM576667
30
G1111_Neocybaeina_burnetti
98
Blabomma_sp_TehamaCounty
98
G3904_Blabomma_californicum
Reduced Eyes Clade
50
MJR2130_Cybaeozyga_furtiva
71
G1114_Cybaeozyga_heterops
12
G4110_Willisus_gertschi
100
G1792_Willisus_gertschi
G1148_Calymmaria_shastae
97
G1436_Calymmaria_persica
74
64
G1087_Siskiyu_gen_nov_WestBeaverCreek
99
G1089_Siskiyu_gen_nov_CaveJunction
84
G0570_Siskiyu_gen_nov_Matthews_Creek
Patellar Fracture Clade
0.04
loci not recovered
G3967_Cryphoeca_exlinae
G4019_Yorima_angelica
G1150_Cybaeina_dixoni

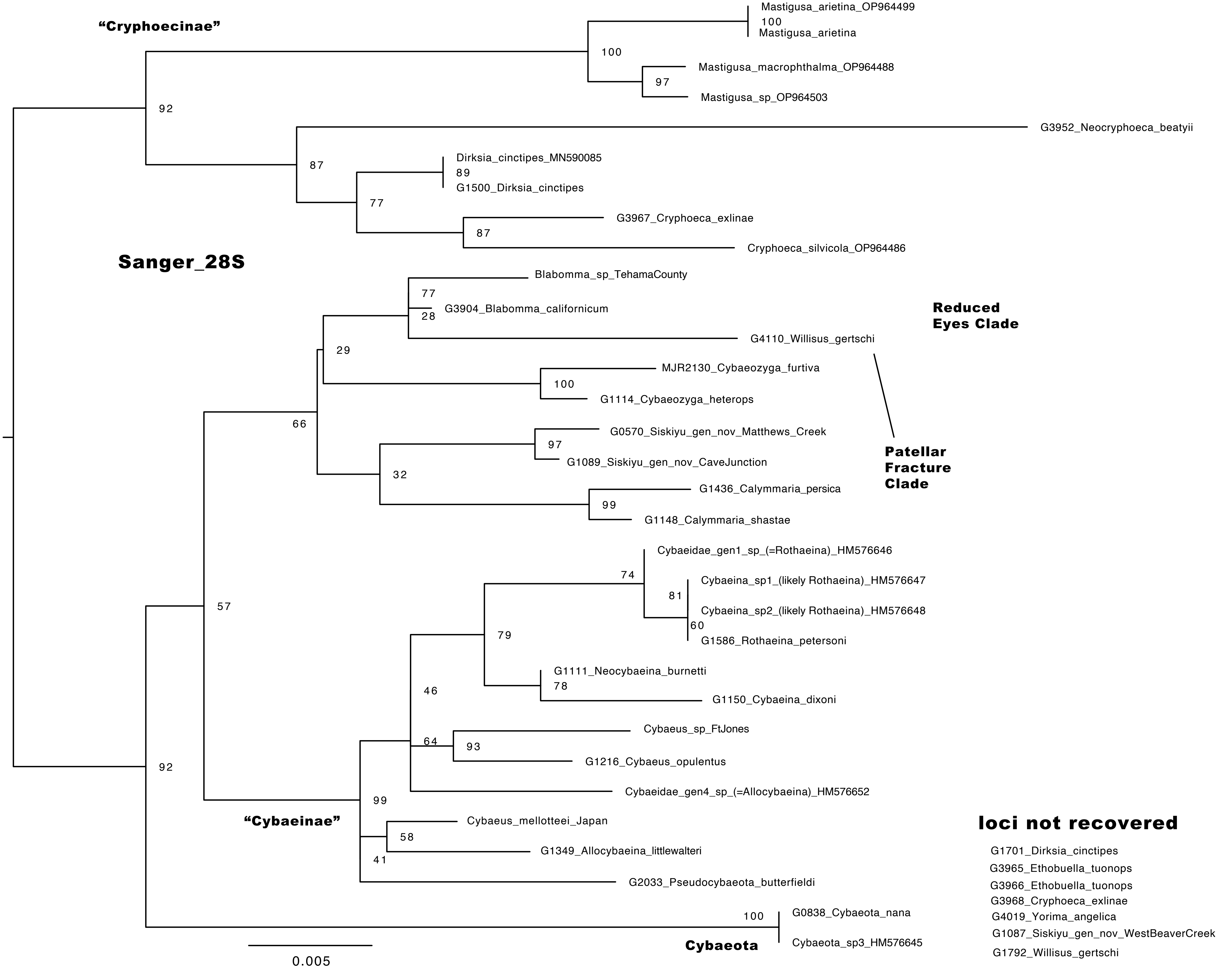

"Cryphoecinae"
Mastigusa_arietina_OP964499
100
Mastigusa_arietina
100
Mastigusa_macrophthalma_OP964488
97
Mastigusa_sp_OP964503
92
G3952_Neocryphoeca_beatyii
87
Dirksia_cinctipes_MN590085
89
G1500_Dirksia_cinctipes
77
G3967_Cryphoeca_exlinae
87
Cryphoeca_silvicola_OP964486
Sanger_28S
Blabomma_sp_TehamaCounty
77
G3904_Blabomma_californicum
28
Reduced Eyes Clade
G4110_Willisus_gertschi
29
MJR2130_Cybaeozyga_furtiva
100
G1114_Cybaeozyga_heterops
66
G0570_Siskiyu_gen_nov_Matthews_Creek
97
G1089_Siskiyu_gen_nov_CaveJunction
Patellar Fracture Clade
32
G1436_Calymmaria_persica
99
G1148_Calymmaria_shastae
Cybaeidae_gen1_sp_(=Rothaeina)_HM576646
74
Cybaeina_sp1_(likely Rothaeina)_HM576647
81
Cybaeina_sp2_(likely Rothaeina)_HM576648
60
G1586_Rothaeina_petersoni
57
79
G1111_Neocybaeina_burnetti
78
46
G1150_Cybaeina_dixoni
Cybaeus_sp_FtJones
64
93
G1216_Cybaeus_opulentus
92
Cybaeidae_gen4_sp_(=Allocybaeina)_HM576652
99
"Cybaeinae"
Cybaeus_mellotteei_Japan
58
G1349_Allocybaeina_littlewalteri
41
G2033_Pseudocybaeota_butterfieldi
100
G0838_Cybaeota_nana
Cybaeota
Cybaeota_sp3_HM576645
0.005
loci not recovered
G1701_Dirksia_cinctipes
G3965_Ethobuella_tuonops
G3966_Ethobuella_tuonops
G3968_Cryphoeca_exlinae
G4019_Yorima_angelica
G1087_Siskiyu_gen_nov_WestBeaverCreek
G1792_Willisus_gertschi

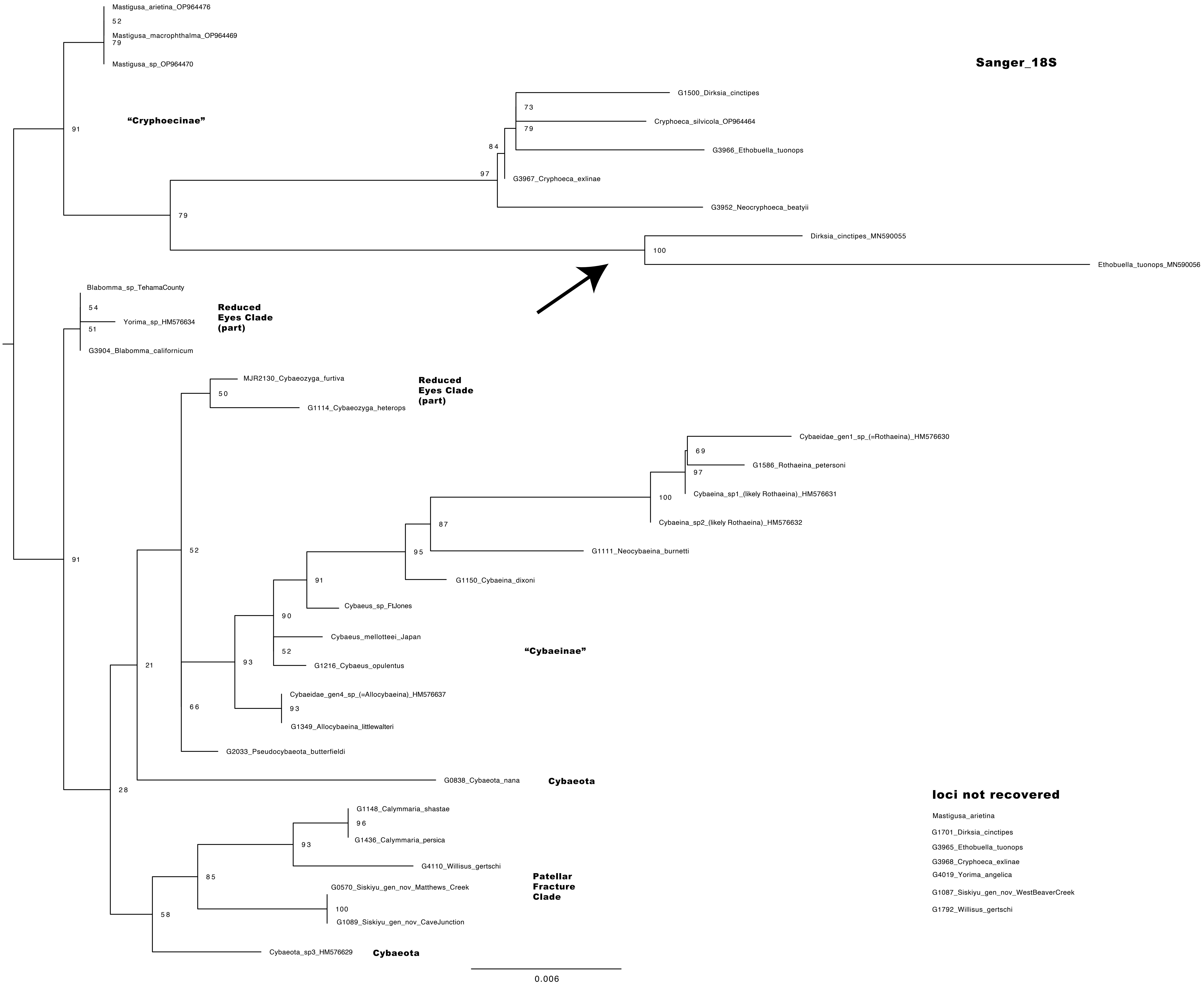
Sanger_18S
Mastigusa_arietina_OP964476
Mastigusa_macrophthalma_OP964469
Mastigusa_sp_OP964470
“Cryphoecinae”
G1500_Dirksia_cinctipes
Cryphoeca_silvicola_OP964464
G3966_Ethobuella_tuonops
G3967_Cryphoeca_exlinae
G3952_Neocryphoeca_beatyii
Dirksia_cinctipes_MN590055
Ethobuella_tuonops_MN590056
Blabomma_sp_TehamaCounty
Yorima_sp_HM576634
G3904_Blabomma_californicum
Reduced Eyes Clade (part)
MJR2130_Cybaeozyga_furtiva
G1114_Cybaeozyga_heterops
Reduced Eyes Clade (part)
Cybaeidae_gen1_sp_(=Rothaeina)_HM576630
G1586_Rothaeina_petersoni
Cybaeina_sp1_(likely Rothaeina)_HM576631
Cybaeina_sp2_(likely Rothaeina)_HM576632
G1111_Neocybaeina_burnetti
G1150_Cybaeina_dixoni
Cybaeus_sp_FtJones
Cybaeus_mellotteei_Japan
G1216_Cybaeus_opulentus
“Cybaeinae”
Cybaeidae_gen4_sp_(=Allocybaeina)_HM576637
G1349_Allocybaeina_littlewalteri
G2033_Pseudocybaeota_butterfieldi
G0838_Cybaeota_nana
Cybaeota
G1148_Calymmaria_shastae
G1436_Calymmaria_persica
G4110_Willisus_gertschi
G0570_Siskiyu_gen_nov_Matthews_Creek
G1089_Siskiyu_gen_nov_CaveJunction
Patellar Fracture Clade
Cybaeota_sp3_HM576629
Cybaeota
0.006
loci not recovered
Mastigusa_arietina
G1701_Dirksia_cinctipes
G3965_Ethobuella_tuonops
G3968_Cryphoeca_exlinae
G4019_Yorima_angelica
G1087_Siskiyu_gen_nov_WestBeaverCreek
G1792_Willisus_gertschi

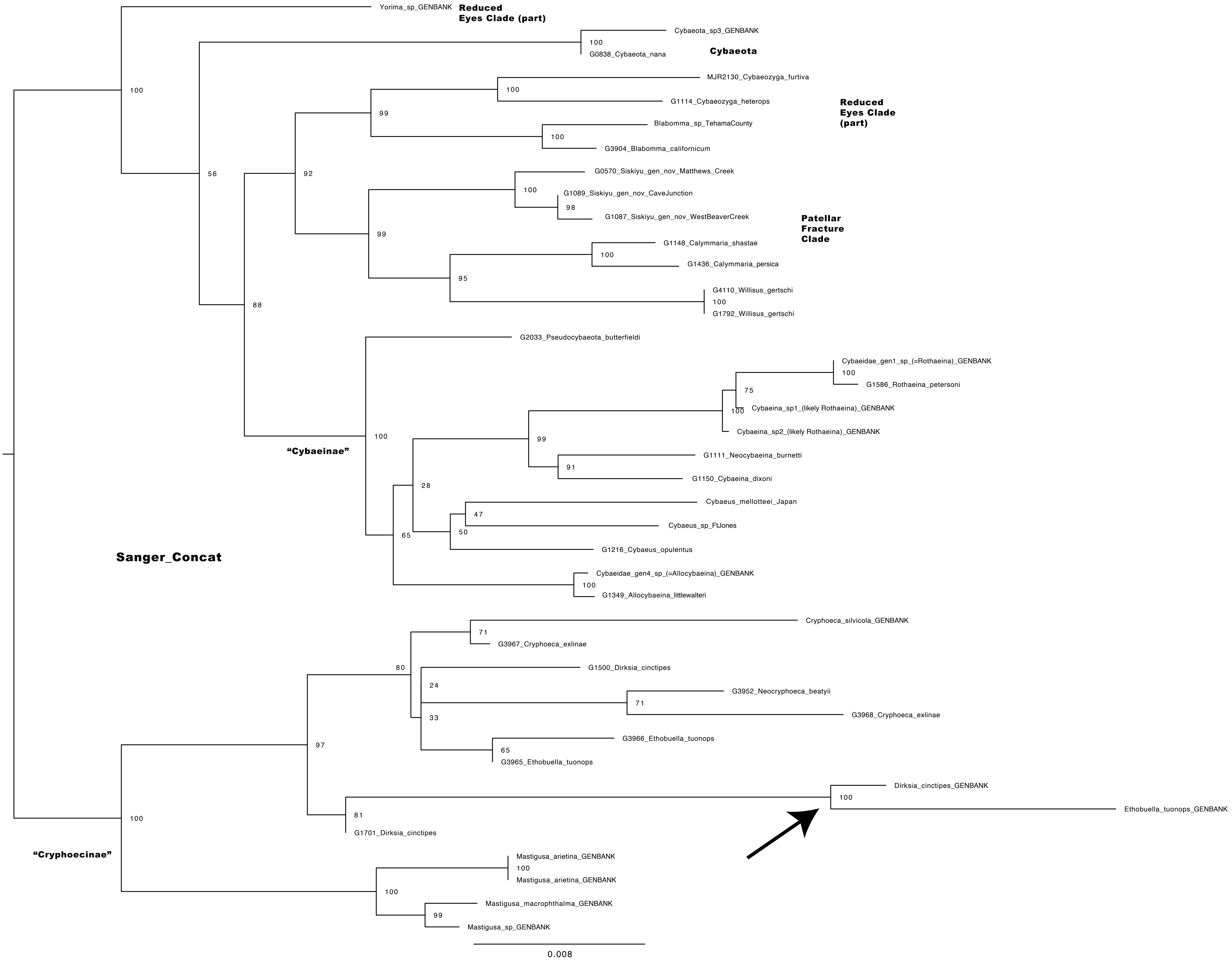

Yorima_sp_GENBANK
Reduced Eyes Clade (part)
Cybaeota_sp3_GENBANK
100
G0838_Cybaeota_nana
Cybaeota
100
MJR2130_Cybaeozyga_furtiva
100
G1114_Cybaeozyga_heterops
Reduced Eyes Clade (part)
99
Blabomma_sp_TehamaCounty
100
G3904_Blabomma_californicum
56
92
G0570_Siskiyu_gen_nov_Matthews_Creek
100
G1089_Siskiyu_gen_nov_CaveJunction
98
G1087_Siskiyu_gen_nov_WestBeaverCreek
Patellar Fracture Clade
99
G1148_Calymmaria_shastae
100
G1436_Calymmaria_persica
95
G4110_Willisus_gertschi
100
G1792_Willisus_gertschi
88
G2033_Pseudocybaeota_butterfieldi
Cybaeidae_gen1_sp_(=Rothaeina)_GENBANK
100
G1586_Rothaeina_petersoni
75
100
Cybaeina_sp1_(likely Rothaeina)_GENBANK
Cybaeina_sp2_(likely Rothaeina)_GENBANK
100
99
"Cybaeinae"
G1111_Neocybaeina_burnetti
91
G1150_Cybaeina_dixoni
28
Cybaeus_mellotteei_Japan
47
Cybaeus_sp_FtJones
50
65
G1216_Cybaeus_opulentus
Sanger_Concat
Cybaeidae_gen4_sp_(=Allocybaeina)_GENBANK
100
G1349_Allocybaeina_littlewalteri
Cryphoeca_silvicola_GENBANK
71
G3967_Cryphoeca_exlinae
80
G1500_Dirksia_cinctipes
24
G3952_Neocryphoeca_beatyii
71
G3968_Cryphoeca_exlinae
33
G3966_Ethobuella_tuonops
97
65
G3965_Ethobuella_tuonops
Dirksia_cinctipes_GENBANK
100
Ethobuella_tuonops_GENBANK
81
100
G1701_Dirksia_cinctipes
"Cryphoecinae"
Mastigusa_arietina_GENBANK
100
Mastigusa_arietina_GENBANK
100
Mastigusa_macrophthalma_GENBANK
99
Mastigusa_sp_GENBANK
0.008
